# Epigenetic silencing of miR-144/451a cluster contributes to HCC progression via paracrine HGF/MIF-mediated TAM remodeling

**DOI:** 10.1186/s12943-021-01343-5

**Published:** 2021-03-03

**Authors:** Junlong Zhao, Huichen Li, Shoujie Zhao, Enxin Wang, Jun Zhu, Dayun Feng, Yejing Zhu, Weijia Dou, Qingling Fan, Jie Hu, Lintao Jia, Lei Liu

**Affiliations:** 1 State Key Laboratory of Cancer Biology, Department of Medical Genetics and Development Biology, Fourth Military Medical University, Xi’an, 710032 China; 2 State Key Laboratory of Cancer Biology, Department of Biochemistry and Molecular Biology, School of Basic Medicine, Fourth Military Medical University, No.169 Changlexi Road, Xi’an, 710032 China; 3 Department of General Surgery, Tangdu Hospital, Fourth Military Medical University, Xi’an, 710038 China; 4 Department of Gastroenterology, Tangdu Hospital of the Fourth Military Medical University, No.569 Xinsi Road, Xi’an, 710038 China; 5 Department of Neurosurgery, Tangdu Hospital, Fourth Military Medical University, Xi’an, 710038 China; 6 Department of Cell Biology, Fourth Military Medical University, No.169 Changlexi Road, Xi’an, 710032 China

**Keywords:** Mir-144, miR-451a, Hepatocellular carcinoma, Tumor-associated macrophage, DNA methylation, Chromosome loop, EZH2

## Abstract

**Background & Aims:**

Hepatocellular carcinoma (HCC) is among the malignancies with the highest mortality. The key regulators and their interactive network in HCC pathogenesis remain unclear. Along with genetic mutations, aberrant epigenetic paradigms, including deregulated microRNAs (miRNAs), exert profound impacts on hepatocyte transformation and tumor microenvironment remodeling; however, the underlying mechanisms are largely uncharacterized.

**Methods:**

We performed RNA sequencing on HCC specimens and bioinformatic analyses to identify tumor-associated miRNAs. The miRNA functional targets and their effects on tumor-infiltrating immune cells were investigated. The upstream events, particularly the epigenetic mechanisms responsible for miRNA deregulation in HCC, were explored.

**Results:**

The miR-144/miR-451a cluster was downregulated in HCC and predicted a better HCC patient prognosis. These miRNAs promoted macrophage M1 polarization and antitumor activity by targeting hepatocyte growth factor (HGF) and macrophage migration inhibitory factor (MIF). The miR-144/miR-451a cluster and EZH2, the catalytic subunit of polycomb repressive complex (PRC2), formed a feedback circuit in which miR-144 targeted EZH2 and PRC2 epigenetically repressed the miRNA genes via histone H3K27 methylation of the promoter. The miRNA cluster was coordinately silenced by distal enhancer hypermethylation, disrupting chromatin loop formation and enhancer-promoter interactions. Clinical examinations indicated that methylation of this chromatin region is a potential HCC biomarker.

**Conclusions:**

Our study revealed novel mechanisms underlying miR-144/miR-451a cluster deregulation and the crosstalk between malignant cells and tumor-associated macrophages (TAMs) in HCC, providing new insights into HCC pathogenesis and diagnostic strategies.

**Supplementary Information:**

The online version contains supplementary material available at 10.1186/s12943-021-01343-5.

## Introduction

Hepatocellular carcinoma (HCC) is among the most common malignant tumors of the digestive system and has a poor prognosis and rising mortality rate [1]. Multiple risk factors, including viral infections, fibrosis, alcohol use, and metabolic dysregulation, induce genetic or phenotypic alterations and contribute to HCC progression [2, 3]. The etiological complexity increased the difficulty of comprehensively understanding the molecular mechanisms of HCC. Over the past decade, new discoveries have established that the immune microenvironment plays pivotal roles in HCC progression [4]. Tumor-associated macrophages (TAMs), which are broadly considered the M2-polarized subtype and suppress antitumor immunity, are involved in the initiation, progression and metastasis of different kinds of HCC [5, 6]. Therapies targeting TAMs have been reported to effectively repress HCC growth in both animal models and clinical trials [5, 7]. Recent strategies have focused on inhibiting TAM generation and re-educating TAMs to M1-like polarization. Interestingly, neoplastic cells also influence the plasticity of TAMs in HCC tissues. Although direct cell contact modulates macrophage development and functions through cell membrane ligands and integrin signaling, accumulated evidence has demonstrated that paracrine factors from HCC cells represent major contributors to immune microenvironment remodeling. HCC-derived cytokines and growth factors such as CCL2, TGF-β, MIF and HGF are required for macrophage recruitment, differentiation and M2-like stimulation [8–11]. It is important to dissect the molecular mechanisms underlying the crosstalk between HCC and TAMs in terms of earlier diagnosis and more efficient intervention.

Epigenetic paradigms, including histone modification, DNA methylation and noncoding RNA (ncRNA) biogenesis, play essential regulatory roles in HCC progression [12]. It has been reported that histone methylation regulates the expression of a subset of proliferation- and metastasis-related genes in HCC [13]. EZH2, the catalytic subunit of the polycomb repressive complex 2 (PRC2) responsible for histone H3K27me3 and subsequent transcriptional repression, is highly expressed in HCC and correlates with the low patient survival rate [14]. EZH2 knockdown or inhibition significantly represses HCC progression [15, 16]. Moreover, DNA methylation by DNA methyltransferases (DNMTs) also plays crucial roles in various stages of HCC development [16, 17]. Tumor suppressor genes are frequently silenced by hypermethylation of CpG islands at the promoter regions in cancer [18]. Indeed, elevated expression of DNMT1 and DNMT3b was associated with poor HCC patient survival [19]. Due to their critical involvement in regulating diverse biological processes and tissue homeostasis, microRNA (miRNA) deregulation has been reported to contribute to the occurrence of various malignancies, including HCC [20, 21]. However, while most studies focus on the downstream events of miRNAs, it is largely elusive how tumor suppressor miRNAs are downregulated in HCC.

In this study, we identified miR-144/miR-451a cluster as a key repressor of HCC development. Further investigation revealed that miR-144/miR-451a promoted M1 polarization of TAMs and enhanced antitumor immunity by targeting hepatocyte growth factor (HGF) and macrophage migration inhibitory factor (MIF). We also discovered a double-negative feedback loop between the miRNA cluster transcription and EZH2-mediated histone modification, and unraveled a detailed mechanism through which DNA methylation-dependent chromatin remodeling regulated miR-144/miR-451a cluster expression in HCC.

## Materials and methods

### Human tissue samples

All HCC and para-tumor tissues were obtained from virus-unrelated HCC patients in the Department of Digestive Surgery, Tangdu Hospital, Fourth Military Medical University. According to WHO guidelines, HCC samples were classified by clinical diagnosis and pathological grading. The study was approved by the Ethics Committee of Fourth Military Medical University and conformed to the Declaration of Helsinki. Informed consent was obtained from all involved patients.

### Mice

C57BL/6 mice were maintained in a specific pathogen-free (SPF) facility. All animal experiments were approved by the Animal Experiment Administration Committee of the Fourth Military Medical University to ensure an ethical and humane treatment of animals.

### RNA sequencing

HCC and adjacent normal tissues were isolated from 7 patients. TAMs of tumor-bearing mice were isolated via FACS. The RNA was extracted from paired samples, and then miRNA and mRNA sequencing were performed with the Illumina HiSeq 3000 at Genergy (Shanghai, China). These data were deposited in GEO with the accession number GSE166349.

### Statistics

Images were imported into Image-Pro Plus 5.1 software (Media Cybernetics, Inc., Bethesda, MA) to quantify the densities of electrophoretic bands. Data were analyzed with GraphPad Prism 5 software. Comparisons between groups were performed with Student’s t-test or one-way ANOVA test. The results are expressed as the mean ± SEM. *P* < 0.05 was considered significant. The detailed descriptions of methods were presented in Supplementary materials.

## Results

### The miR-144/miR-451a cluster suppresses HCC development and predicts better HCC prognosis

**Fig. 1:**
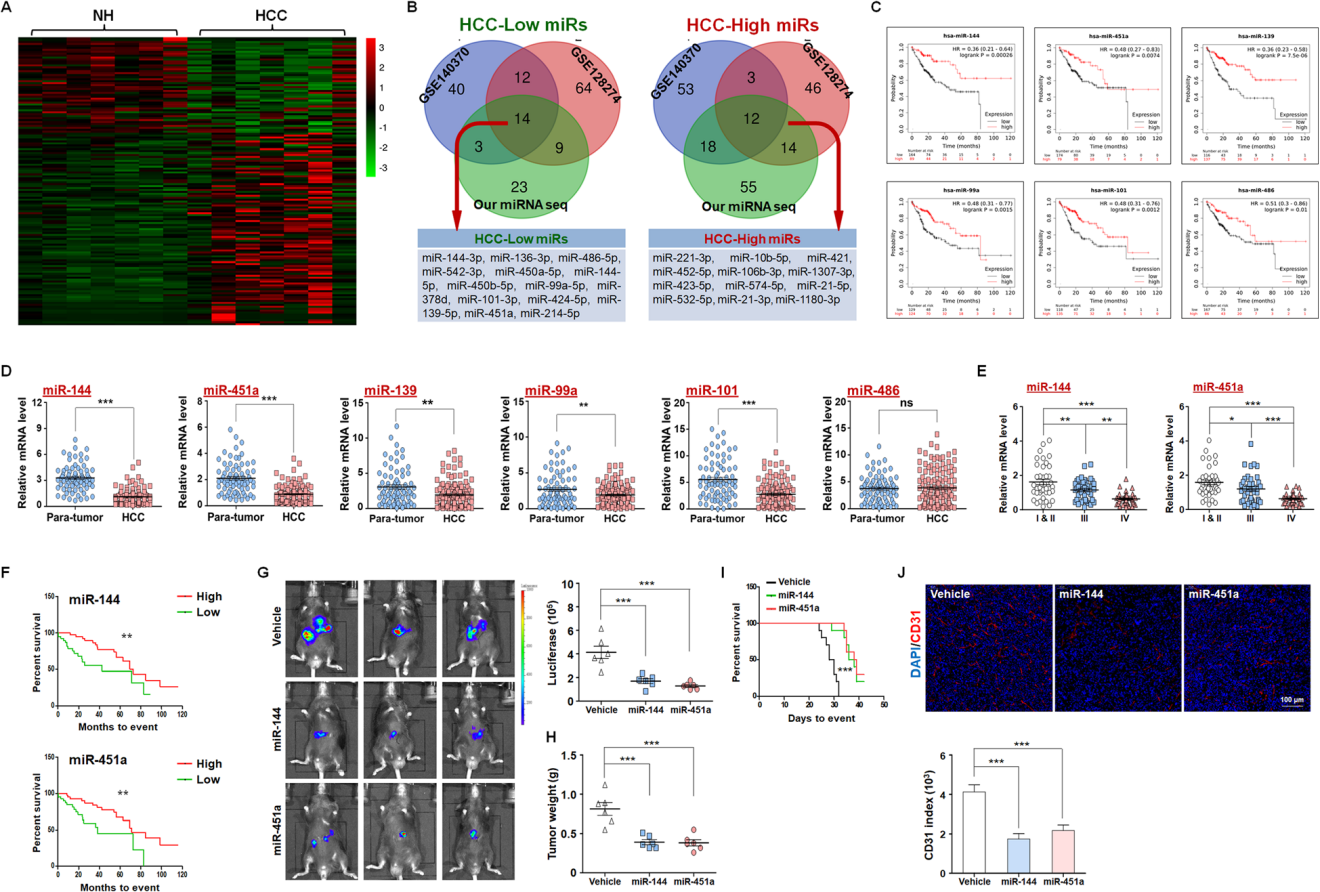
The miR-144/miR-451a cluster correlates with HCC prognosis and polarization of microenvironmental macrophages. **a** Normal hepatic tissues and HCC were isolated from HCC patients, and miRNA expression was profiled by using miRNA sequencing (*n* = 7). **b** Three sets of miRNA expression data were compared, and the common differentially expressed HCC-low and HCC-high miRNAs are presented in a Venn diagram. **c** The relationship between the expression of candidate miRNAs and HCC patient prognosis was evaluated using the TCGA database (*n* = 253). **d** The expression of candidate miRNAs was determined in para-tumor and HCC tissues (*n* = 125). **e** The expression of miR-144 and miR-451a was analyzed in HCC with pathological grade I/II (*n* = 40), grade III (*n* = 40) and grade IV (*n* = 45). **f** HCC patients were grouped according to the levels of miR-144 or miR-451a in tumor tissues and were subjected to survival analysis (*n* = 125). **g-j** Luciferase-expressing Hepa1–6 cells were infected with control or miR-144/miR-451a lentiviruses and inoculated intrahepatically into C57BL/6 mice. Tumor development was evaluated by bioluminescence imaging 3 weeks after the injection (*n* = 6) (**g**). Tumor tissues were excised and weighed after mice were sacrificed (*n* = 6) (**h**). Kaplan-Meier survival analysis was performed for tumor-bearing mice in different groups (*n* = 6) (**i**). CD31 staining by immunofluorescence was performed to detect tumor angiogenesis (*n* = 6) (**j**). Bars, means ± SEMs; *, *P* < 0.05; **, *P* < 0.01; ***, *P* < 0.001

MicroRNAs (miRNAs), which mature from the primary transcript after sequential processing by the ribonucleases Drosha and Dicer, are well documented regulators of carcinogenesis [22]. We first screened for miRNAs involved in the pathogenesis of HCC. Analysis using a TCGA dataset revealed that increased expression of the miRNA-producing enzyme, Dicer1, predicted a better prognosis of HCC (Fig. S1A). Knockdown of Dicer1 in hepatoma Hepa1–6 cells promoted transplanted tumor formation by these cells in isogenic C57BL/6 mice (Fig. S1B-D). Sequencing of miRNAs in 7 pairs of HCC and adjacent NH tissues detected significant alterations in miRNA expression profiles during carcinogenesis (Fig. 1a). These results were combined with published datasets in the Gene Expression Omnibus (GEO, GSE128274 and GSE140370) to identify potential tumor suppressor miRNAs and oncogenic miRNAs in HCC (Fig. 1b and Fig. S2A, B). The consistently altered miRNAs in HCC patients from TCGA database then underwent Kaplan-Meier survival analysis. As a result, we identified 13 miRNAs that correlated with patient prognosis, of which 6 were downregulated and 7 upregulated in HCC (Fig. 1c and Fig. S2C, D). These miRNAs included several documented key players in HCC [23–25]. Further analysis using 125 paired clinical specimens validated that 5 of the 6 candidate tumor suppressors in HCC tissues displayed reduced expression compared with those in para-tumor tissues (Fig. 1d). Of note were miR-144 and miR-451a, which are transcribed from the same locus of both human (chr17) and mouse (chr11) chromatin with an interval less than 100 bp. Indeed, these 2 miRNAs were coordinately expressed in hepatic cell lines and tissues of both human and mouse origins, with consistently decreased levels in malignant cells or tissues (Figs. S3 and S4). In line with the aforementioned analysis based on published datasets, we found that both miRNAs were associated with early tumor staging and better prognosis of tested HCC patients (Fig. 1e, f).

To evaluate the antitumor function of miR-144/miR-451a in vivo, Hepa1–6 cells, which were modified to constitutively express luciferase, were inoculated intrahepatically into C57BL/6 mice. Three weeks after challenging, tumor development was evaluated by bioluminescence imaging, which indicated that miR-144 or miR-451a overexpression in Hepa1–6 cells impeded in vivo tumor development from these cells (Figs. 1g, S5A and B). Although the body weights were indistinguishable among these groups, tumor and liver weights decreased when miR-144/miR-451a was overexpressed in Hepa1–6 cells, and miR-144 or miR-451a overexpression prolonged the survival of HCC-bearing mice (Fig. 1h, i). In addition, CD31 staining suggested that miR-144 or miR-451a also repressed tumor angiogenesis (Fig. 1j). We also transfected miR-144/miR-451a in another mouse HCC cell line, H22, followed by inoculation subcutaneous inoculation in the right back of recipient mice. As a result, overexpression of miR-144 or miR-451a significantly repressed tumor growth (Figs. S5C, D). Thus, miR-144 and miR-451a are clustered miRNAs that play repressive roles in HCC pathogenesis.

### miR-144/miR-451a in HCC cells promotes M1-like polarization and the antitumor effect of macrophages

**Fig. 2:**
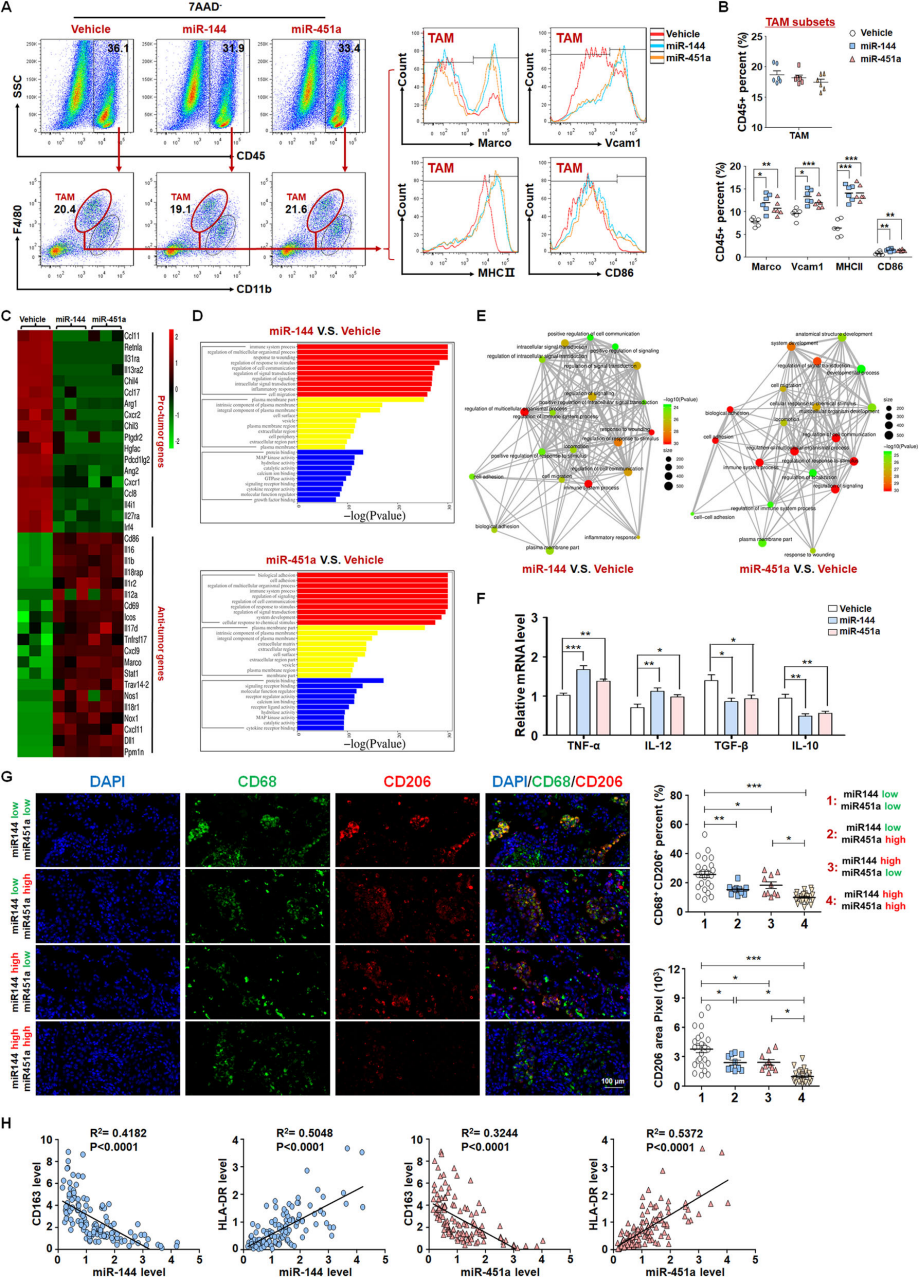
miR-144/miR-451a modulates the HCC paracrine function to promote macrophage M1-like polarization. **a, b** Control Hepa1–6 cells or those overexpressing miR-144 or miR-451a were inoculated intrahepatically into C57BL/6 mice. Tumors were then dissected, FACS was performed to analyze cell subsets expressing pan-macrophage and polarized macrophage markers (*n* = 6). **c-f** TAMs were sorted for RNA-seq. The differentially expressed genes related with inflammation were compared (**c**) and analyzed for functional clusters (D, E) (*n* = 3). The mRNA levels of macrophage polarization markers in sorted TAM subsets from (**c**) were assayed using qRT-PCR (F, *n* = 6). **g, h** The tumor tissues of HCC patients were grouped according to miR-144/miR-451a expression and stained for M2-polarized marker CD206 (**g**) (1: *n* = 25; 2: *n* = 10; 3: *n* = 10; 4: *n* = 25). The relationships between miR-144/miR-451a expression and the levels of CD68, CD163 or HLA-DR were analyzed (*n* = 125) (**h**). Bars, means ± SEMs; *, *P* < 0.05; **, *P* < 0.01; ***, *P* < 0.001

We next investigated whether miR-144/miR-451a suppress HCC progression via directly inhibiting the growth or invasion of neoplastic cells. As shown in Fig. S6, these miRNAs failed to significantly repress the malignant phenotypes of mouse hepatoma Hepa1–6 cells, suggesting that they function via cancer cell non-autonomous mechanisms. Immunosuppressive tumor microenvironment is one of the cancer hallmarks and plays important roles in tumor initiation and progression. FACS assay of orthotopic HCC derived from inoculated Hepa1–6 cells indicated that miR-144/miR-451a overexpression improved the ratios of infiltrating CD8^+^ T cells and reduced Tregs, but didn’t affect the subsets of B cells, NK/NKT or myeloid-derived suppressor cells (MDSCs) (Fig. S7A-E). TAMs, typically resembling M2-polarized phenotypes of macrophages, are the key cells that facilitate immune escape of malignant cells [26]. We found in orthotopic tumors that overexpression of miR-144/miR-451a did not affect the percentages of CD11b^+^F4/80^high^ TAMs (Fig. 2a). However, miR-144 or miR-451a promoted the expression of M1-like macrophage markers Marco, Vcam1, MHC II and CD86 in tumor tissues (Fig. 2b). Furthermore, TAMs were next sorted from orthotopic HCC of different groups for RNA-seq, which showed that anti-tumor cytokines or molecules were increased in TAMs from miR-144 or miR-451a overexpression groups (Fig. 2c). Functional analysis of the cluster further indicated that pathways of immune system process and inflammatory response were altered remarkably in TAMs (Fig. 2d, e). Quantitative RT-PCR assay indicated that TAMs from miR-144- or miR-451a-overexpressing tumors showed increased M1-type and decreased M2-type cytokine production (Fig. 2f). The remodeling of TAMs and activation of cytotoxic T cells after overexpression of miR-144/miR-451a in HCC cells were also manifested using another in vivo tumor model derived from H22 cells (Fig. S8A-D).

We next analyzed the role of miR-144/miR-451a in TAM polarization using clinical HCC samples. Co-staining for CD68, a pan-marker for macrophages, with the M1-polarized macrophage marker NF-κB p65 or the M2 marker CD206, was performed and compared among the groups. The results indicated that CD206^+^ TAMs were prevalent in miR144^low^miR451a^low^ HCC tissues, while miR144^high^miR451a^high^ HCCs showed rare staining for M2-like CD206^+^ TAMs (Fig. 2g). Conversely, p65^+^CD68^+^ macrophages were found in much higher densities in miR144^high^miR451a^high^ HCCs (Fig. S9). Examination of the clinical HCC specimens showed that miR-144 and miR-451a were negatively correlated with the M2-polarized macrophage marker CD163, and positively correlated with the M1-polarized macrophage marker HLA-DR (Fig. 2h) [26]. These data suggest that high levels of miR-144/miR-451a in HCC facilitate M1 polarization of tumor microenvironmental TAMs.

TAMs polarization is regulated by cytokines, chemokines and inflammatory mediators derived mainly from tumor cells [27]. We thus investigated whether miR-144/miR-451a-expressing neoplastic cells promote M1 polarization of TAMs via paracrine factors. We incubated the supernatant of miR-144/miR-451a-overexpressing HCC cells with BMDMs. The results showed that miR-144 or miR-451a overexpression in Hepa1–6 cells generated media that promoted M1 polarization of BMDMs, as revealed by an increased production of M1-type cytokines and nitric oxide (NO) when untreated or subjected to LPS/IFN-γ stimulation for M1 polarization (Fig. S10A-C); expression of miR-144 or miR-451a in HCC cells also impaired the BMDM response to stimuli for M2 polarization since both the secretion of M2-type cytokines and the expression of CD206 and arginase 1 were repressed by these miRNAs (Fig. S10A, B, D). The function of BMDMs was further evaluated through coculture with CFSE-stained Hepa1–6 cells (Fig. S10E). The results showed that BMDMs cultured with supernatants from miR-144/miR-451a-overexpressing Hepa1–6 cells displayed significantly higher basal and LPS/IFN-γ-induced phagocytic activity than those cultured with supernatants prepared from control Hepa1–6 cells (Fig. S10F, G). We next assessed the ability of macrophages to activate T cells via coculture and subsequently measured the cytolytic activity of T cells (Fig. S10H). As a result, BMDMs cultured with supernatants from miR-144/miR-451a-overexpressing Hepa1–6 cells exhibited a stronger capability to prime T cells than those cultured with supernatants from control Hepa1–6 cells (Fig. S10I). Conversely, inhibition of miR-144 or miR-451a in Hepa1–6 cells by anti-sense oligonucleotides (ASOs) suppressed the response of BMDMs to M1 polarization stimuli, although their M2 polarization remained largely unchanged (Fig. S11A-C). Therefore, miR-144/miR-451a in HCC cells promotes M1 polarization and the antitumor function of macrophages via a paracrine pathway.

### miR-144 and miR-451a confer paracrine activation of macrophage M1 polarization by targeting HGF and MIF

**Fig. 3:**
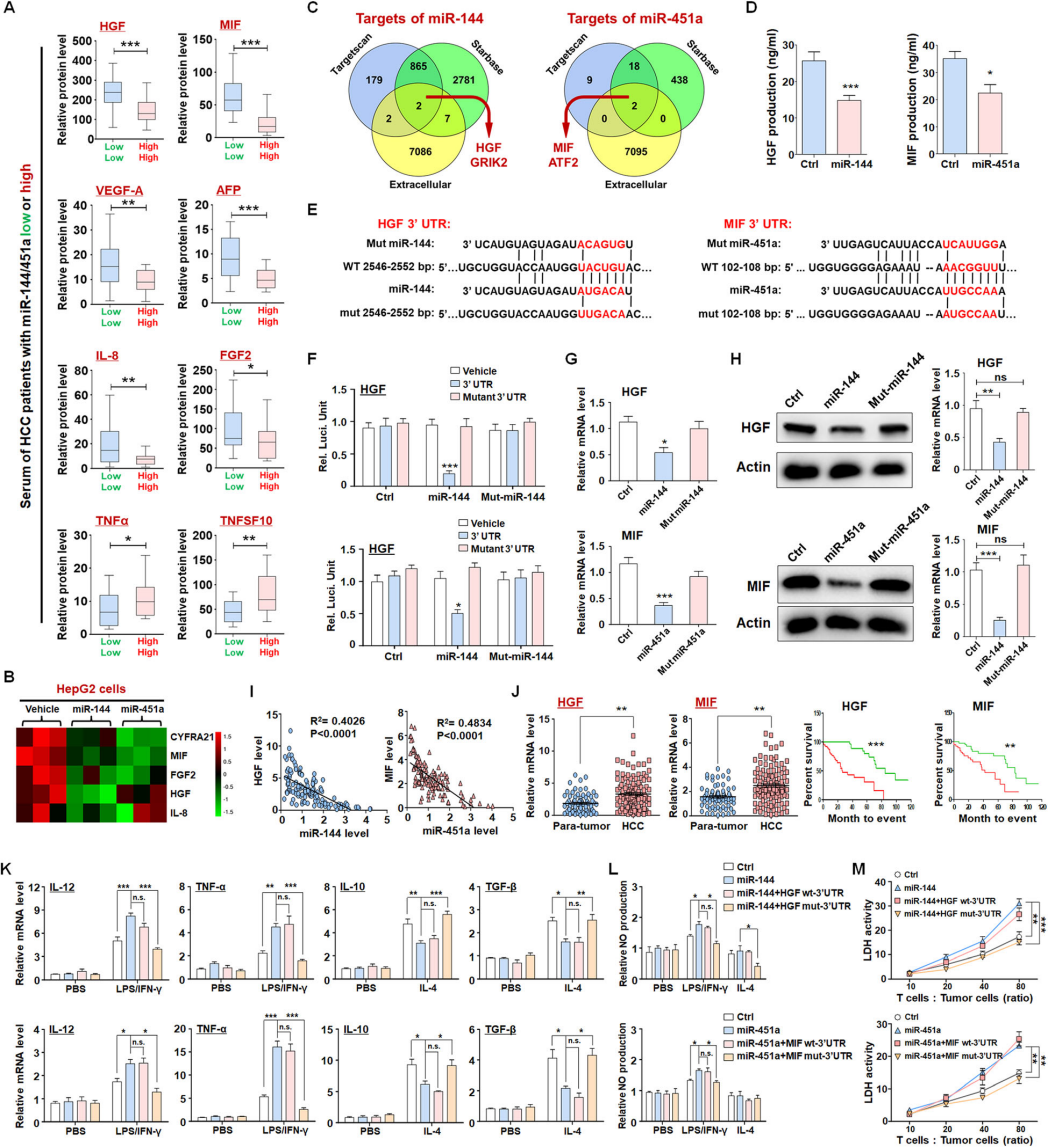
miR-144/miR-451a regulate macrophage polarization by targeting HGF and MIF in HCC cells. **a, b** The abundances of secreted cytokines were determined by cytokine antibody array using sera of HCC patients (**a**) (*n* = 36) and supernatant of control HCC cells or those overexpressing miR-144 or miR-451a (**b**) (*n* = 3). **c** The targets of miR-144 or miR-451a were predicted using different online tools and classified according to the protein subcellular localization. The extracellular target proteins were selected. **d** The production of HGF and MIF was detected in control and miR-144/miR-451a-overexpressing HepG2 cells using ELISA (*n* = 5). **e** Bioinformatic prediction identified HGF as a target of miR-144 and MIF as a target of miR-451a. **f** Reporter assay was performed to validate that miR-144 inhibited luciferase expression from a construct harboring a 3’UTR of *HGF* or *MIF* (*n* = 6). **g, h** Hepa1–6 cells were subjected to lentivirus-mediated overexpression of miR-144 or seed sequence-mutated miR-144/miR-451a, followed by qRT-PCR (**g**) and Western blot (**h**) analyses for the expression of HGF or MIF (*n* = 6). **i** The correlation between the levels of HGF mRNA and miR-144 or the levels of MIF and miR-451a in clinical HCC samples was analyzed (*n* = 125). **j** Paired clinical para-tumor and HCC tissues were used to determine the levels of HGF and MIF via qRT-PCR (*n* = 125). HCC patients were grouped according to the levels of HGF or MIF in tumor tissues and were subjected to survival analysis (*n* = 125). **k, l** miR-144- or miR-451a-overexpressing Hepa1–6 cells were further modified to express *HGF* or *MIF* with WT 3’UTR or the miRNA-binding site-mutated 3’UTR, and the supernatant was prepared and used for culture of BMDMs. The expression of polarization markers (**k**) (*n* = 6) and the generation of NO (**l**) by BMDMs were determined. **m** BMDMs cultured in conditioned media as described in (**k**) were further cocultured with CD3^+^ T cells. The activated T cells were then cocultured with Hepa1–6 cells at different ratios of cell numbers. Bars, means ± SEMs; *, *P* < 0.05; **, *P* < 0.01; ***, *P* < 0.001

To fully understand how miR-144/miR-451a-mediated paracrine signaling regulated macrophages in HCC, cytokine antibody array was performed to determine the abundance of cancer-secreted cytokines in sera of miR144^low^miR451a^low^ and miR144^high^miR451a^high^ HCC patients. As the data shown, 6 cytokines were significantly higher and 2 cytokines lower in miR144^high^miR451a^high^ HCC among the Cancer Biomarker Panel (including 23 cytokines) (Figs. 3a and S12). We also detected the cytokines in supernatant of HCC cells, in which the levels of 5 cytokines were altered by overexpression of miR-144 or miR-451a (Figs. 3b and S13). The production of HGF, MIF and IL-8 was changed consistently in the sera of HCC patients and supernatants of HCC cells. We employed bioinformatic algorithms (TargetScan and StarBase) and extracellular database (https://www.proteinatlas.org/) to predict the targets of miR-144 and miR-451a [28, 29]. As shown in Fig. 3c, HGF and GRIK2 were potential targets of miR-144, and MIF and ATF2 were predicted as targets of miR-451a. Validation by ELISA suggested that miR-144 and miR-451a suppressed the secretion of HGF and MIF, respectively (Fig. 3d). Therefore, HGF and MIF are the candidate targets of miR-144/miR-451a cluster in HCC. A reporter assay showed that miR-144 suppressed luciferase expression of the transcript containing the wild-type 3′-UTR of *HGF* but not that of the transcript containing the seed sequence-mutated 3′-UTR (Fig. 3e, f). In addition, miR-144 overexpression in HepG2 cells reduced both the mRNA and protein levels of HGF (Fig. 3g, h), whereas miR-144 inhibition by ASOs enhanced HGF expression (Fig. S14A, B). The regulation of miR-451a on target MIF was also confirmed by luciferase reporter assay (Fig. 3j), qRT-PCR (Figs. 3g and S14C) and Western blot detection (Figs. 3h and S14D). Consistent with these observations, miR-144 and miR-451a overexpression also inhibited the levels of both tumor and serum HGF and MIF in mouse HCC derived from Hepa1–6 or H22 cells (Fig. S14E-G). The levels of miR-144 and miR-451a were inversely correlated with those of HGF and MIF, respectively, in clinical HCC samples (Fig. 3i). These results suggest that HGF and MIF are direct targets of miR-144 and miR-451a in HCC cells.

HGF was reported to promote M2 polarization of macrophages [30] and MIF established to account for the immunosuppressive role of TAMs [31]. We thus reasoned that miR-144/miR-451a in HCC cells regulate macrophage polarization through affecting the secretion of HGF and MIF. We found that the levels of HGF and MIF were much higher in HCC cells than in normal hepatocytes (Fig. S15). HGF and MIF were also upregulated in HCC tissues compared with those in para-tumor tissues, and predicted poor prognosis of HCC patients (Fig. 3j). In addition, the HGF receptor, c-Met, and the MIF receptor, CD74, were both expressed on BMDMs and TAMs (Fig. S16A). Next, Hepa1–6 cells overexpressing miR-144 were further transfected with constructs of HGF with wild-type (WT) or the predicted miR-144 binding site-mutated 3’UTR, respectively. The supernatants of these cells were then collected and used for culture of BMDMs. In contrast to those cultured in media from miR-144-overexpressing Hepa1–6 cells, BMDMs incubated with media from cells transfected with a mutant 3’UTR-containing HGF construct, but not that with a WT 3’UTR, exhibited reduced M1-like and enhanced M2-like polarization, as indicated by the cytokine expression and NO production levels comparable to the characteristics of cells cultured in control media (Fig. 3k, l). In addition, miR-144 inhibition by ASO in Hepa1–6 cells promoted M2 polarization of BMDMs, which was completely reversed by an antibody of HGF (Fig. S16B). Similarly, MIF transcripts with mutant 3’UTR but not that with WT 3’UTR in Hepa1–6 cells rescued the effects of miR-451a on BMDM polarization (Fig. 3k, l). The function of miR-451a ASO was also counteracted by an MIF antibody (Fig. S16C). Media prepared from cells transfected with mutants of miR-144 or miR-451a disabled for binding to the mRNAs of HGF and MIF were comparable to those from control cells in terms of the regulation of BMDM polarization (Fig. S17). The increase in the capacity of macrophages to activate cocultured T cells, as observed upon miR-144 or miR-451a expression in Hepa1–6 cells, was also ablated by further overexpression of HGF or MIF with mutant 3’UTR but not that with WT 3’UTR (Fig. 3m). The paracrine regulation of macrophages by neoplastic cells mediated by miR-144/miR-451a was also confirmed with cell lines of human HCC (HepG2 and Huh7) and a human macrophage cell line, THP1 (Fig. S18). These data suggest that miR-144/miR-451a in HCC cells promotes M1-like and represses M2-like polarization of environmental macrophages by targeting HGF and MIF, respectively.

### miR-144 and miR-451a form a negative feedback regulatory circuit with EZH2

**Fig. 4:**
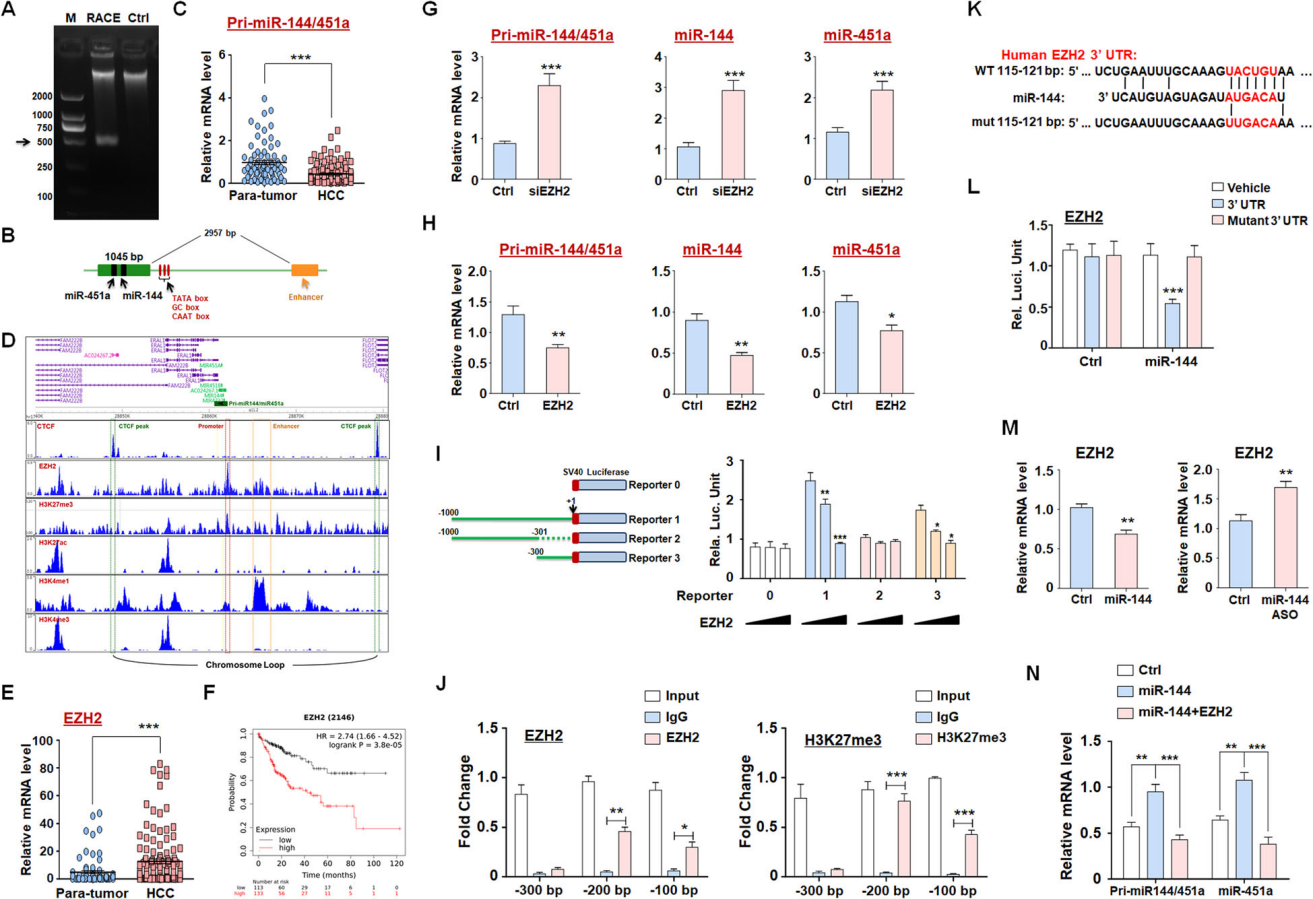
miR-144/miR-451a and EZH2 form a regulatory circuit in HCC cells. **a** 5′-RACE was performed to identify the full-length transcript from the pri-miR-144/451a cluster. The amplified fragment (664 bp) is indicated by the arrow. **b** The structural information of pri-miR-144/451a is presented as a diagram, which contains one exon (green box), an enhancer region (E1, orange box) and several typical transcription-related boxes (red stick). **c** The expression of this primary miRNA was examined in para-tumor and HCC tissues (*n* = 125). **d** The potential epigenetic modifications in the promoter and enhancer regions of pri-miR-144/451a were analyzed using the EZH2, H3K27me3, H3K27ac, H3K4me1 and H3K4me3 ChIP-seq data of hepatocytes or HCC cells from the Cistrome Project. **e** The levels of EZH2 in clinical para-tumor and HCC tissues were measured (*n* = 125). **f** The relationship between EZH2 expression and HCC patient prognosis was evaluated using data from TCGA (*n* = 253). **g, h** HepG2 cells were modified to overexpress EZH2 (**g**) or subjected to EZH2 knockdown (**h**), followed by qRT-PCR assay for the expression of pri-miR-144/451a, miR-144 and miR-451a (*n* = 6). **i** Luciferase constructs for truncated fragments of the pri-miR-144/451a promoter were generated, and reporter assays were performed to determine the binding regions of EZH2 on the pri-miR-144/451a promoter (*n* = 6). 0, vehicle; 1, − 1000 bp to + 1 bp; 2, − 1000 bp to − 300 bp; 3, − 300 bp to + 1 bp. **j** ChIP assays were performed using HepG2 cell lysates to further determine the pri-miR-144/451a promoter sites involved in EZH2 binding and H3K27me3 modification (*n* = 4). **k, l** Bioinformatic prediction identified EZH2 as a target of miR-144 (**k**), which was validated by an assay showing that miR-144 inhibited luciferase expression of a construct harboring a 3’UTR of *EZH2* (**l**) (*n* = 6). **m** The mRNA level of EZH2 was assayed in miR-144-overexpressing or miR-144-inhibited HepG2 cells (*n* = 6). **n** HepG2 cells were modified to overexpress miR-144 or to synchronously express miR-144 and EZH2. The expression of pri-miR-144/451a and miR-451a was detected via qRT-PCR (*n* = 6). Bars, means ± SEMs; *, *P* < 0.05; **, *P* < 0.01; ***, *P* < 0.001

To investigate the regulation of miR-144/miR-451a expression in HCC, we first performed rapid amplification of 5′ cDNA ends (5′-RACE) to determine the transcription start site (TSS) of pri-miR-144/451a using cDNAs generated from HepG2 cells. A fragment of approximately 500 bp was amplified (Fig. 4a), which was sequenced and mapped to a chromatin region with several typical transcription-related elements close to the TSS (Fig. 4b). Analysis using clinical specimens showed that the putative pri-miR-144/451a transcript in HCC tissues was downregulated compared with that in para-tumor tissues (Fig. 4c). In addition, ChIP-seq data with HCC cells from the Cistrome Project (http://cistrome.org/) suggested high level of H3K27me3 and low levels of H3K4me3, HeK4me1 and H3K27ac near the TSS of pri-miR-144/451a in HCC (Fig. 4d). Interestingly, hepatocytes presented lower level of H3K27me3 in promoter of pri-miR-144/451a compared with HCC cells (Fig. S19). Histone H3K27 methylation, which is commonly associated with epigenetic gene silencing, is predominantly mediated by PRC2 [32]. Consistent with previous findings [33], we observed that EZH2, the catalytic subunit of PRC2, in HCC tissues was upregulated compared with that in adjacent normal tissues, and EZH2 expression correlated with poor prognosis of HCC patients (Fig. 4e, f). EZH2 overexpression dramatically reduced the levels of pri-miR-144/451a and both mature miRNAs in HepG2 cells, whereas EZH2 knockdown significantly upregulated these miRNA levels (Fig. 4g, h), suggesting that they were silenced at least partially by histone H3K27me3 of the promoter in HCC. To determine the exact promoter regions involved in EZH2-mediated pri-miR-144/451a silencing, we generated luciferase reporter constructs for truncated 5′ flanking sequences and found that − 300 bp upstream of the TSS in the proximal promoter was indispensable for EZH2 repression of transcription (Fig. 4i). ChIP assays further verified that EZH2 bound to and induced histone H3K27me3 of the − 200 to 0 bp region on the pri-miR-144/451a promoter (Fig. 4j). Interestingly, EZH2 is also a reported target of miR-144, which was validated here by a luciferase reporter assay and the observation that miR-144 overexpression and inhibition reduced and increased EZH2 levels, respectively, in HepG2 cells (Fig. 4k-m) [34]. Finally, ectopic miR-144 expression induced the upregulation of both pri-miR-144/451a and miR-451a in HepG2 cells, which was abolished by overexpression of EZH2 (Fig. 4n). Together, these data demonstrate that miR-144/451a and EZH2 form a regulatory circuit in HCC cells.

### Enhancer methylation induces chromatin remodeling and contributes to pri-miR-144/451a repression in HCC

**Fig. 5:**
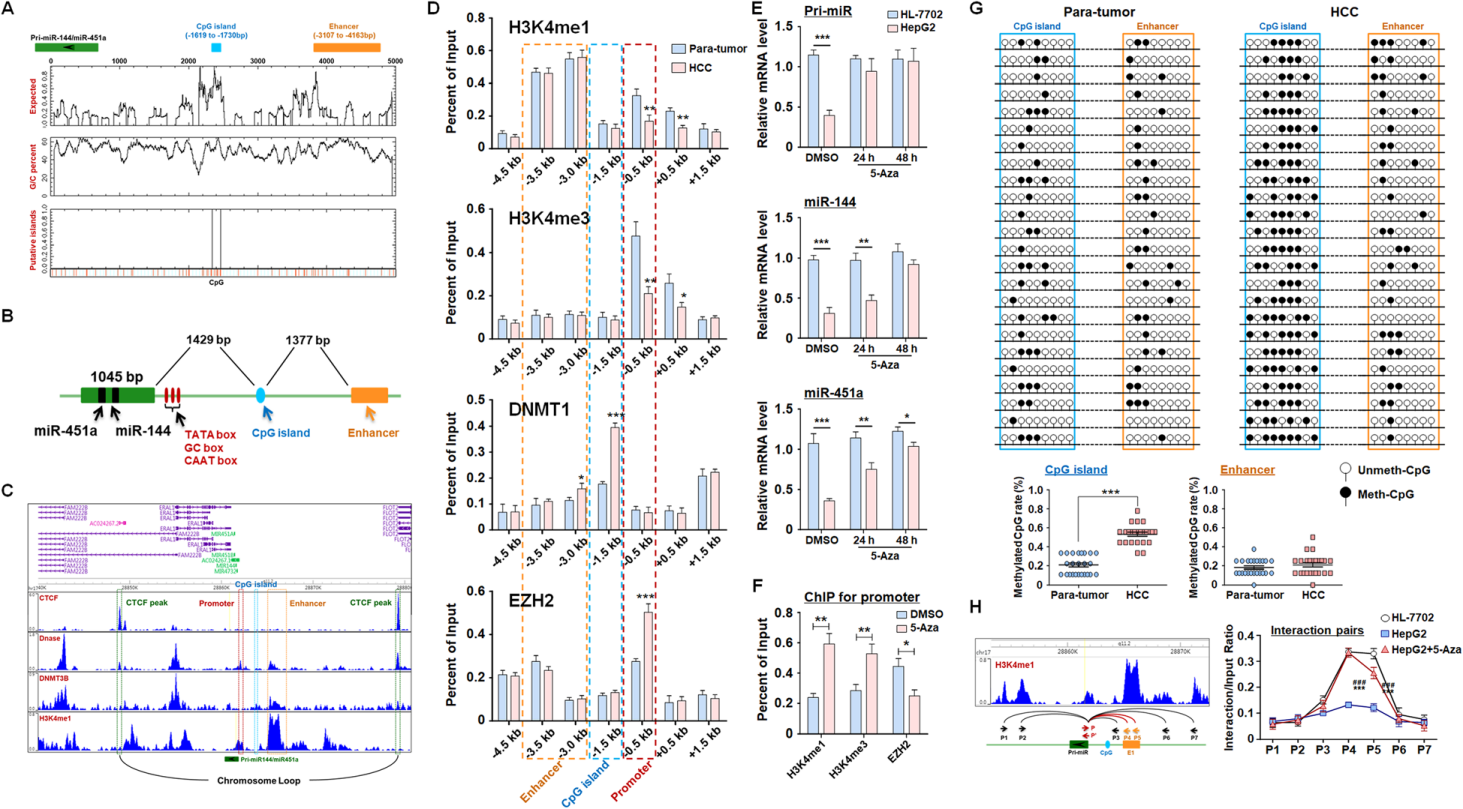
The methylation of upstream CpG islands represses pri-miR-144/451a expression by impairing enhancer-promoter interaction in HCC cells. **a** The sequence of a chromosome region encompassing the pri-miR-144/451a locus and the 5′-flanking sequences was analyzed, and the CpG islands were predicted. **b** A diagram showing the relative position of pri-miR-144/451a, the predicted CpG island and the E1 enhancer region. **c** ChIP-seq data from the Cistrome Project were used to analyze sites potentially bound by CTCF, DNase and DNMT1 on the pri-miR-144/451a locus. **d** ChIP assays were performed using lysates of para-tumor and HCC tissues. Different fragments of the pri-miR-144/miR-451a promoter were amplified and compared between tumor and para-tumor tissues (*n* = 7). **e** The normal hepatic cell line HL-7702 and HCC line HepG2 were treated with a DNMT1 inhibitor (5-Aza, 5 μM) or DMSO. The expression of the indicated miRNA transcripts was determined via qRT-PCR (*n* = 5). **f** HepG2 cells were treated with 5-Aza or DMSO and ChIP assays were performed by antibodies of H3K4me1, HeK4me3 and EZH2. The region of pri-miR-144/451a promoter was amplified (*n* = 4). **g** The DNA methylation levels on the predicted intronic CpG island and the promoter of pri-miR-144/451a in para-tumor and HCC tissues were evaluated via BSP (*n* = 24). **h** A 3C assay was performed to detect the interaction between the E1 region (orange arrows) and other different regulatory elements (black arrows) within the pri-miR-144/451a promoter (red arrows). Bars, means ± SEMs; *, *P* < 0.05; **, *P* < 0.01; ***, *P* < 0.001

Although the pri-miR-144/451a promoter was inactivated in HCC cells, an active enhancer (E1) was located close to the TSS of pri-miR-144/451a (3107 bp upstream) (Fig. 4b and d). CCCTC-binding factor (CTCF) is a ubiquitously expressed and highly conserved 11-zinc finger protein that mediates the formation of chromatin loops, which usually allow transcription by satisfying the interaction between promoters and enhancers [35]. The pri-miR-144/451a locus and E1 are between two strong CTCF-binding peaks, raising the possibility that a chromatin loop encompassing E1 and the promoter region licenses the transcription of pri-miR-144/451a (Fig. 4d). In addition, a CpG island was predicted to be located upstream of the TSS of pri-miR-144/451a between the enhancer and proximal promoter (Fig. 5a, b). ChIP-seq data from the Cistrome Project indicated that this CpG island could be recognized by DNA methyltransferase 1 (DNMT1), the key enzyme that maintains chromatin DNA methylation (Fig. 5c).

We next performed ChIP assays using paired HCC and para-tumor tissues to probe existing epigenetic modifications in these 5′-flanking regions of the pri-miR-144/451a locus. The comparable H3K4me1 levels suggest no difference in the E1 enhancer activity between malignant and adjacent normal tissues; however, the promoter activity of pri-miR-144/451a was much lower in HCC than in adjacent normal tissues, as evidenced by remarkably reduced H3K4me3 levels and elevated EZH2 levels (Fig. 5d). In concert with these observations, we detected improved DNMT1 enrichment in the aforementioned CpG island region in HCC (Fig. 5d). These results were in agreement with the ChIP assays using an HCC cell line, HepG2, and a normal hepatic cell line, HL-7702 (Fig. S20). Treatment of HepG2 cells with a DNMT inhibitor, 5-azacytidine (5-Aza), increased the levels of primary or mature miR-144/451a (Fig. 5e) and promoter activity of the miRNA cluster (Fig. 5f). The epigenetic regulation mechanism of miR-144/miR-451a was verified in mouse HCC cells, which indicated the miR-144/EZH2 circuit could regulate the expression of miR-144/miR-451a by affecting H3K27me3 of the promoter (Fig. S21A-E). Meanwhile, the conserved CpG island located upstream of miR-144/miR-451a insulated the miRNAs cluster from the enhancer in HCC (Fig. S21F-H). Binding of DNMT1 on the CpG island synchronized with reduced promoter activity in HCC cells (Fig. S21I), which was restored by a DNMT1 inhibitor (Fig. S21J). These data suggest that the silencing of pri-miR-144/451a in HCC was dependent on DNMT1-catalyzed methylation on the CpG island.

We next measured the methylation of this CpG island in clinical HCC samples and found that it was hypermethylated in HCC but not in para-tumor tissues (Fig. 5g). Finally, we evaluated the effect of CpG island methylation on the potential interaction between E1 and the promoter of pri-miR-144/451a using a 3C assay. As shown in Fig. 5h, potent interactions between the pri-miR-144/451a promoter and E1 (P4 and P5) regions were detected in HL-7702 and 5-Aza-treated HepG2 cells but not in untreated HepG2 cells. These results demonstrate that hypermethylation of the CpG island blocks the promoter-enhancer interaction, thereby contributing to the repression of pri-miR-144/451a in HCC.

### Deletion of the upstream CpG island promoted miR-144/miR-451a expression and repress HCC growth by modulating TAM polarization

To validate the regulatory role of the upstream CpG island and the enhancer in miR-144/miR-451a expression, we deleted these regions in Hepa1–6 cells via the CRISPR/cas9 system. The CpG island deletion (ΔCpG) increased miR-144/miR-451a and decreased HGF and MIF expression, while the enhancer deletion (ΔEnh) exhibited the opposite effect (Fig. S22A, B). The supernatant of ΔCpG or ΔEnh HCC cells was then incubated with macrophages, which indicated that ΔCpG supernatant promoted M1 polarization and phagocytic activity of macrophages, while ΔEnh supernatant stimulated M2 polarization and attenuated engulfment function of macrophages (Fig. S22C, D).

**Fig. 6:**
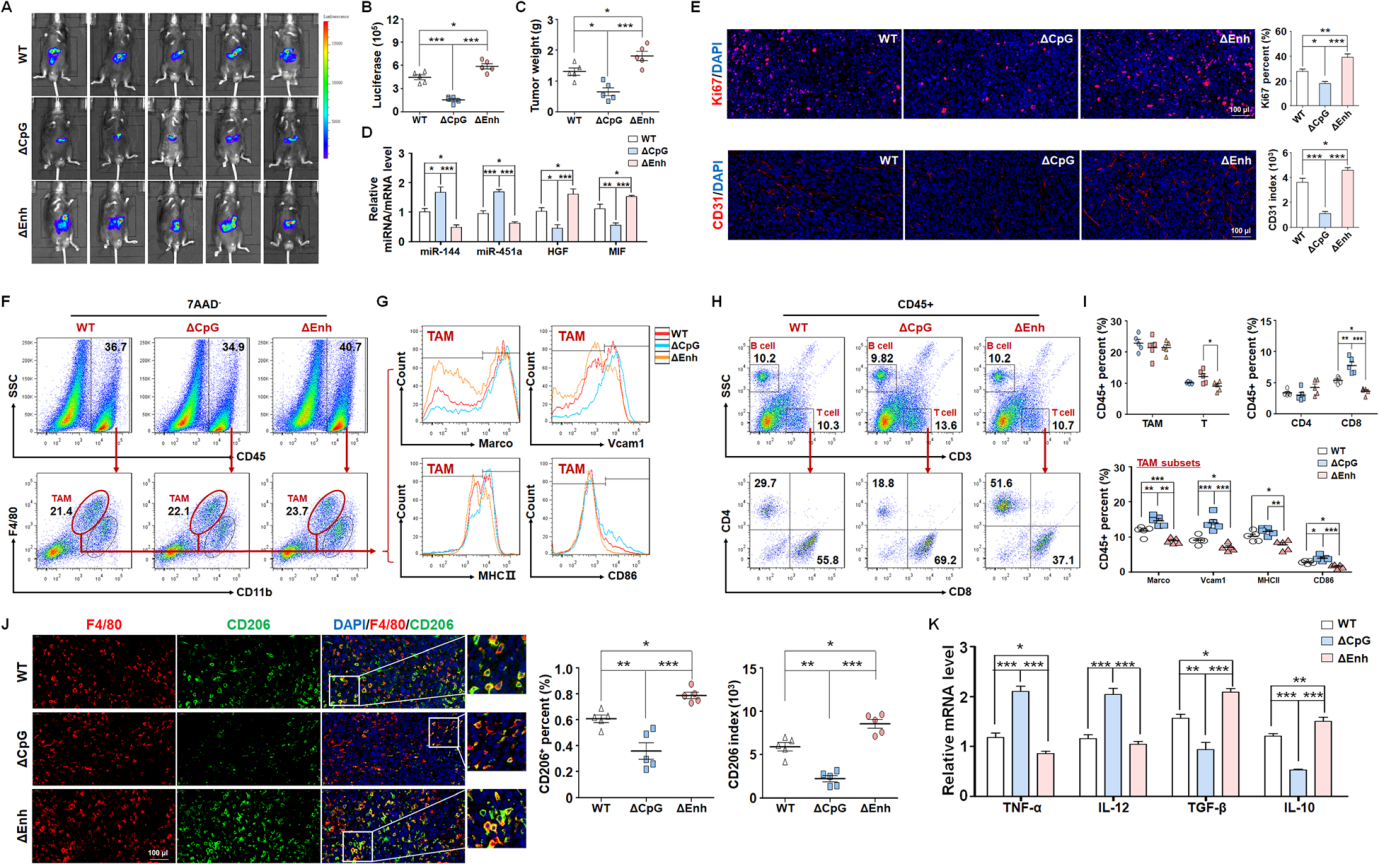
miR-144/451a upstream CpG island or enhancer deletion modulates HCC progression in vivo by regulating macrophage polarization. **a, b** The CpG island or enhancer region upstream to miR-144/451a was deleted via CRISPR/cas9 system in luciferase-expressing Hepa1–6 cells, and cells were inoculated intrahepatically into C57BL/6 mice. Tumor development was evaluated by bioluminescence imaging 3 weeks after the injection (*n* = 5). **c** The tumor tissues were excised and weighed after mice were sacrificed (*n* = 5). **d** The expression of miR-144/miR-451a and their targets in tumor tissues from different groups in (**a**) was measured via qRT-PCR (*n* = 5). **e** Immunofluorescence staining for Ki67 and CD31 was performed to assess tumor cell proliferation and angiogenesis (*n* = 5). **f-i** FACS was performed to analyze the subsets and differentiation of TAMs (**f, g**) and phenotype of lymphocytes (**h**) from different groups, and the cell percentages were plotted and compared (**i**) (*n* = 5). **j** The tumor tissues were co-stained with F4/80 and CD206 and analyzed for expression of the M2-like macrophage marker, CD206 (*n* = 5). **k** The mRNA levels of macrophage polarization markers in sorted TAMs were measured via qRT-PCR (*n* = 5). Bars, means ± SEMs; *, *P* < 0.05; **, *P* < 0.01; ***, *P* < 0.001

Next, the ΔCpG or ΔEnh HCC cells were inoculated intrahepatically into C57BL/6 mice, which showed that the CpG island depletion retarded tumor growth and removal of the enhancer accelerated HCC progression (Fig. 6a-c). qRT-PCR assay using the tumor tissues manifested that CpG or the enhancer deletion affected the expression of the miRNA cluster and their targets, which was consistent with the in vitro examinations (Fig. 6d). Ki67 and CD31 staining indicated that CpG island deficiency reduced while the enhancer depletion elevated tumor cells proliferation and angiogenesis (Fig. 6e). Furthermore, macrophages in the tumor microenvironment were investigated. Compared with the parental HCC cells, ΔCpG showed increased whereas ΔEnh exhibited reduced M1-like polarization of TAMs development and differentiation CD8^+^ cytotoxic T cells (Fig. 6f-i). Staining for F4/80 and CD206 as well as qRT-PCR assay using sorted TAMs validated that M2-like polarization of TAMs was attenuated and accelerated by deletion of the CpG island and the enhancer, respectively (Fig. 6j, k). Blocking antibodies of HGF and MIF ablated the effects of ΔEnh on the development of Hepa1–6 cell-derived mouse tumors and the polarization of infiltrating macrophages (Fig. S23A-E). Taken together, our findings elucidated that DNA methylation-dependent chromatin remodeling regulated miR-144/miR-451a cluster expression and further controlled the plasticity of TAMs.

## Discussion

HCC is an intricate disease ascribed to multiple causative aspects and risk factors [36]. Unlike many malignancies arising from definitive driving genetic mutations or signaling pathways, the molecular pathogenesis of HCC is extremely complex and heterogeneous, highlighting the importance of a nonlinear regulatory network and epigenetic mechanisms in carcinogenesis [36, 37]. miRNAs play key roles in HCC by orchestrating the expression of multiple genes involved in parallel or interactive signaling events [38]. In the present study, we systemically analyzed the miRNA profiles in HCC by combining our sequencing results of clinical specimens with published datasets, and the validity of these datasets was demonstrated by the identification of previously reported critical miRNAs regulating HCC progression [23–25]. Of these key miRNAs deregulated in HCC, we established that the miR-144/−451a cluster correlated with a better prognosis of clinical HCC. Mechanistically, hepatocellular miR-144/miR-451a aroused the antitumor immunity of infiltrating macrophages via paracrine signaling; miR-144/miR-451a also constituted a feedback circuit with EZH2, the core histone H3K27 methyltransferase known to promote HCC pathogenesis by epigenetically silencing the tumor suppressor genes. Unlike other studies reporting that miR-144/miR-451a suppress proliferation of HCC cells by targeting oncogenes like EZH2 [39, 40], we found that these miRNAs exert a tumor-suppressive role mainly through remodeling the tumor immune microenvironment. These discrepancies might be attributed to the heterogeneity of HCC or the use of different experimental systems including cells with distinct gene expression profiles or intracellular signaling contexts. While other miRNAs have been reported to participate in this epigenetic paradigm [24, 41], miR-144/miR-451a might exert more potent effects on HCC since they target both the autonomous machinery of neoplastic cells and the microenvironment hijacked to support tumorigenesis.

The progression of solid tumors, including HCC, relies on the establishment of an immunosuppressive microenvironment characterized by a massive accumulation of tumor-promoting myeloid immune cells, especially TAMs [42]. When confronting environmental stimuli, macrophages undergo polarization into M1-type macrophages, which play important roles in the inflammatory response and antitumor immunity, or M2-type macrophages, which are crucially involved in tissue repair and tumor progression [43]. TAMs are widely accepted as a macrophage population mainly displaying the M2-like phenotype [44]. In addition to classical stimuli (e.g., LPS and IFN-γ for M1 and IL-10 and TGF-β for M2 polarization), the differentiation of macrophages is also determined by redundant factors from parenchymal cells [43]. In particular, HGF promotes M2 polarization of macrophages, thereby participating in the anti-inflammatory response in various tissues and facilitating tumor progression [30]. Recently, MIF has been reported as a determinant of M2-like activation of macrophages [45]. We found that miR-144/miR-451a overexpression impaired the M2 phenotype and stimulated M1-like polarization of macrophages by targeting and reducing the secretion of HGF and MIF from HCC cells, resulting in elevated phagocytosis and an enhanced capability to activate cytotoxic T lymphocytes. Compared with exosome-delivered miRNAs, miRNA-mediated paracrine factor secretion might represent a pattern of intercellular crosstalk with high efficiency and cell-type specificity [46]. Although there are contradicting reports about the cellular origin of TAMs in the tumor microenvironment, we observed the activation of TAMs in both intrahepatic and subcutaneous tumors by miR-144/miR-451a-overexpressing HCC cells [47]. Nonetheless, considering the high macrophage plasticity, further investigations are warranted to determine whether this activation necessarily includes the repolarization of the tumor cell-educated M2 population to an M1-like phenotype [42].

Accumulating evidence has shown that epigenetic gene regulation requires coordinated changes, including covalent DNA and histone modifications and chromatin conformational remodeling, in chromosomal loci [48]. We found that miR-144/451a, as a cluster, is transcriptionally repressed by EZH2-catalyzed histone H3K27 methylation of the promoter in HCC cells. Moreover, we identified an active enhancer (E1) in the 5′-flanking regions, the CTCF elements of which enable a potential interaction between E1 and the promoter. As expected, we verified an existing loop covering these chromatin regions in normal hepatocytes. Interestingly, the formation of this chromatin loop was dependent on the hypomethylation of an embedded CpG island, suggesting that simultaneous histone H3K27 methylation of the promoter and DNA methylation of the distal enhancer are required to silence miR-144/451a in HCC cells. When the CpG island or E1 enhancer was deleted, the expression of miR-144/451a, the polarization of TAMs and the progression of orthotopic tumors were all influenced. While DNA methylation represents the best characterized form of epigenetic modification, the detailed mechanisms leading to gene repression may vary substantially in different genomic sites, e.g., the formation of heterochromatin, impeded access to transcriptional activators, and recruitment of transcription-repressive complexes [49, 50]. Our findings on the miR-144/451a locus revealed the pivotal involvement of chromatin remodeling in DNA methylation-elicited gene silencing and highlighted coordinated DNA and histone modifications in epigenetic gene regulation. Together, these data provide novel insights into the regulatory roles of noncoding RNAs in HCC pathogenesis and have implications for the feasibility of targeting multiple epigenetic components for the clinical treatment of HCC.

## Conclusions

Our findings identify miR-144/miR-451a cluster as a tumor suppressor in HCC, which induce M1-like repolarization of TAMs though paracrine pathway via targeting HGF and MIF. In a regulatory circuit, miR-144 targets EZH2 and EZH2-catalyzed histone H3K27 methylation silences the miR-144/miR-451a cluster in HCC. Additionally, it is the first time to unveil that the CpG island upstream to miR-144/miR-451a cluster drives the chromatin conformation remodeling in HCC patients, providing new insights into HCC pathogenesis and diagnostic strategies.

## Supplementary Information

**Additional file 1: Supplementary Materials and Methods**. **Table S1.** Primers and oligonucleotides used in this study. **Table S2.** Antibodies used in this study. **Figure S1.** Dicer1 suppresses HCC development and correlates with a better prognosis of HCC patients. (A) The relationship between the expression of Dicer1 and the prognosis of male virus-unrelated HCC patients was evaluated using data from TCGA (*n* = 96). (B-D) Hepa1-6 cells after Dicer1 knockdown via vector-based shRNA transfection were used for subcutaneous inoculation of C57BL/6 mice. Mice were sacrificed four weeks after inoculation, and tumors were excised for examination (*n* = 5). Bars, means ± SEMs; **, *P* < 0.01. **Figure S2.** Correlation of miRNAs with HCC prognosis in published datasets. (A, B) The miRNA expression profiles in published GEO datasets (GSE140370, *n* = 3; and GSE128274, *n* = 4). (C, D) The relationship between the expression of candidate miRNAs and HCC patient prognosis was evaluated using these datasets (*n* = 253). **Figure S3.** The relationship of miR-144 and miR-451a expression in HCC (*n* = 125). **Figure S4.** The expression of miR-144 and miR-451a is repressed in human and mouse HCC cell lines and tissues. (A-D) The expression of miR-144 and miR-451a was measured via qRT-PCR in normal hepatocyte (NH) and HCC cell lines of human (A and B) and mouse (C, D) organs (*n* = 6). (E, F) Hepa1-6 cells were inoculated intrahepatically into C57BL/6 mice, and paratumor and HCC tissues were isolated to measure the expression of miR-144 and miR-451a (*n* = 5). Bars, means ± SEMs; **, *P* < 0.01; ***, *P* < 0.001. **Figure S5.** miR-144 or miR-451a overexpression suppresses HCC development in vivo. (A, B) Control Hepa1-6 cells or those overexpressing miR-144 or miR-451a were inoculated intrahepatically into C57BL/6 mice. Body weight and liver weight were examined three weeks after the injection (*n* = 6) (A). Tumors were then dissected, and the expression of miR-144/miR-451a was measured via q-RT-PCR (*n* = 6) (B). (C, D) H22 cells were infected with control or miR-144/miR-451a-overexpressing lentiviruses and were subcutaneously inoculated on C57BL/6 mice. Tumor tissues were excised and weighed three weeks after the inoculation (*n* = 5). The expression of miR-144/miR-451a was measured via q-RT-PCR (*n* = *n* = 5) (D). Bars, means ± SEMs; **, *P* < 0.01; ***, *P* < 0.001. **Figure S6.** miR-144 or miR-451a had no effect on proliferation and apoptosis of HCC cells. Hepa1-6 cells were infected by control or miR-144/miR-451a-overexpressing lentiviruses. Cells were then subject to CCK-8 assay (A, *n* = 5), FACS assay for cell cycle pregression (B, *n* = 5) and apoptosis (C, *n* = 5). The expressions of epithelial and mesenchymal markers were also examined via qRT-PCR (D, *n* = 5). Bars, means ± SEM. **Figure S7.** miR-144/miR-451a overexpression affects microenvironmental cell subsets in an orthotopic HCC model. (A-E) Control Hepa1-6 cells or those overexpressing miR-144 or miR-451a were inoculated intrahepatically into C57BL/6 mice. Tumors were then dissected, and FACS was performed to analyze the subsets of lymphocytes (A-D) and MDSCs (E) (*n* = 6). Bars, means ± SEMs; **, *P* < 0.01; ***, *P* < 0.001. **Figure S8.** miR-144/miR-451a overexpression modulates the phenotypes of microenvironmental cells in an orthotopic HCC model derived from H22 cells. (A-D) H22 cells were infected with control or miR-144/miR-451a-overexpressing lentiviruses and were subcutaneously inoculated on C57BL/6 mice. The tumor tissues were excised, and FACS was performed to analyze the subsets and differentiation of TAMs (A) and phenotype of lymphocytes (B) from different groups, and the cell percentages were plotted and compared (C) (*n* = 5). The mRNA levels of macrophage polarization markers were detected using qRT-PCR in sorted TAMs (D, *n* = 5). Bars, means ± SEMs; *, *P* < 0.05; **, *P* < 0.01; ***, *P* < 0.001. **Figure S9.** The miR-144/miR-451a cluster correlates with M1-polarization of microenvironmental macrophages. (A, B) The tumor tissues of HCC patients were stained with F4/80 and p65 (A) and analyzed for expression of indicated markers (B) (1: *n* = 25; 2: *n* = 10; 3: *n* = 10; 4: *n* = 25). **Figure S10.** miR-144/miR-451a modulates the HCC paracrine function to promote macrophage M1-like polarization and antitumor effects *in vitro*. (A-D) BMDMs were cultured from bone marrow cells via M-CSF stimulation. Hepa1-6 cells were infected by recombinant lentiviruses to overexpress miR-144 or miR-451a, and the supernatant was prepared to culture BMDMs with the indicated stimuli. The function of macrophages was determined by qRT-PCR (A, *n* = 6), ELISA (B, *n* = 6) and Western blot (D, n =4) assays for the expression of the indicated genes or measurement of NO generation (C, *n* = 8). (E-G) BMDMs were incubated in the supernatant of Hepa1-6 cells as described in (A) and then cocultured with CFSE-stained Hepa1-6 cells (E). Differentiated macrophages were identified by staining for F4/80, and tumor cell engulfment by macrophages was detected via FACS (F, G) (n= 6). (H, I) BMDMs were incubated with the supernatant of Hepa1-6 cells as described in (A) and then cultured for 3 days with CD3+ T cells sorted from the spleen by magnetic beads. The activated T cells were cocultured with Hepa1-6 cells at different ratios of cell numbers. The cytotoxicity of tumor cells was assessed by LDH activity determination (*n* = 6). Bars, means ± SEMs; *, *P* < 0.05; **, *P* < 0.01; ***, *P* < 0.001. **Figure S11.** Inhibition of miR-144 or miR-451a in HCC cells represses macrophage M1-like polarization via the paracrine pathway. BMDMs were cultured from bone marrow cells via M-CSF stimulation. Hepa1-6 cells were transfected with ASOs of miR-144 or miR-451a, and the supernatant was collected for culturing BMDMs, which were further subjected to polarization stimulation with the indicated factors. The function of macrophages was determined by measuring the expression of cytokines via qRT-PCR (A), production of these cytokines via ELISA (B) and generation of NO (C) (*n* = 5). Bars, means ± SEMs; *, *P* < 0.05; **, *P* < 0.01. **Figure S12.** The abundances of the cancer secreted cytokines were determined by cytokine antibody array from serum of HCC patients (*n* = 36). Bars, means ± SEMs. **Figure S13.** The abundances of the cancer secreted cytokines which showed no difference among supernatant of Hepa1-6 cells with different treatment by cytokine antibody array (*n* = 3). **Figure S14.** miR-144 and miR-451a inhibit the expression of HGF and MIF, respectively, both in vitro and in vivo. (A, B) Hepa1-6 cells were subjected to ASO-mediated inhibition of miR-144 or seed sequence mutated miR-144 (Mut-miR-144), followed by qRT-PCR (A) and Western blot (B) analyses for the expression of HGF (*n* = 6). (C, D) Hepa1-6 cells were subjected to ASO-mediated inhibition of miR-451a or seed sequence mutated miR-451a (Mut-miR-451a) followed by qRT-PCR (C) and Western blot (D) analyses for the expression of MIF (*n* = 6). (E) Hepa1-6 cells were infected with control or miR-144/miR-451a lentiviruses and inoculated intrahepatically into C57BL/6 mice. The tumor tissues were excised three weeks after the inoculation, and the expression of HGF and MIF was examined viaqRT-PCR. (F, G) H22 cells were infected with control or miR-144/miR-451a-overexpressing lentiviruses and were subcutaneously inoculated on C57BL/6 mice. Tumor tissues were excised and the serum was collected three weeks after inoculation. HGF and MIF mRNA levels in tumor tissues (F) and protein levels in the serum (G) were examined via qRT-PCR and ELISA, respectively (*n* = 5). Bars, means ± SEMs; *, *P* < 0.05; **, *P* < 0.01; ***, *P* < 0.001. **Figure S15.** qRT-PCR assay for the expression of HGF and MIF in normal hepatic (NH) and HCC cell lines (*n* = 6). **Figure S16.** miR-144/451a in HCC cells regulate macrophage polarization through paracrine HGF and MIF. (A) Monocytes and TAMs were sorted from bone marrow (BM) and tumor tissue, respectively. BMDMs were stimulated from BM cells with M-CSF (25 ng/ml) for 7 days. The expression of HGF receptor (c-Met) and MIF receptor (CD74) was determined in these cells. (B) BMDMs were cultured from bone marrow cells via M-CSF stimulation. Hepa1-6 cells were transfected with miR-144 ASO or/and incubated with HGF antibody (αHGF), and the supernatant was used for culture of stimulated BMDMs. The expression of indicated genes in macrophages was determined by qRT-PCR (*n* = 5). (C) Hepa1-6 cells were transfected with miR-451a ASO or/and incubated with MIF antibody (αMIF), and the supernatant was used for culture of stimulated BMDMs. The expression of indicated genes in macrophages was determined by qRT-PCR (*n* = 5). Bars, means ± SEMs; *, *P* < 0.05; **, *P* < 0.01; ***, *P* < 0.001; ns, not significant. **Figure S17.** Mutant miR-144/miR-451a expression in HCC cells fails to affect the polarization of cocultured BMDMs. Hepa1-6 cells were modified to overexpress seed sequence-mutated miR-144 (Mut-miR-144) or mutated miR-451a (Mut-miR-451a), and the supernatant was prepared to culture BMDMs with the indicated stimuli. The function of macrophages was determined by qRT-PCR (*n* = 5). Bars, means ± SEMs. **Figure S18.** miR-144/miR-451a in human HCC cells regulate the function of cocultured human macrophages by targeting HGF and MIF. (A, B) HepG2 (A) and Huh7 (B) cells were modified to overexpress miR-144 or miR-451a, and human recombinant HGF (20ng/ml) or MIF (20ng/ml) was added as indicated. The supernatant was harvested and used for culture of stimulated human macrophage cell line, THP1 (*n* = 5). (C, D) THP1 cells were cultured as described in (A, B), and the NO generation was measured (*n* = 5). Bars, means ± SEMs; *, *P* < 0.05; **, *P* < 0.01; ***, *P* < 0.001. **Figure S19.** The potential H3K27me3 modification in the promoter and enhancer regions of pri-miR-144/451a were analyzed using ChIP-seq data of hepatocytes or HCC cells from the Cistrome Project. **Figure S20.** Enhanced DNMT1 enrichment and regressed promoter activity on the pri-miR-144/451a locus in HCC cell lines. (A-C) ChIP assays were performed using lysates of HepG2 cells with antibodies of H3K4me1, H3K4me3 and DNMT1. The different fragments of the Pri-miR-144/miR-451a promoter were amplified and compared between the two cell lines. **Figure S21.** DNA methylation-dependent chromatin remodeling regulates miR-144/451a cluster in mouse HCC cells. (A, B) Mouse EZH2 was also identified as a target of mouse miR-144, which was validated by an assay showing that miR-144 inhibited luciferase expression from a construct harboring a 3’UTR of mouse *EZH2* (*n* = 6). (C) The mRNA level of EZH2 was detected in miR-144-overexpressing or miR-144-inhibited Hepa1-6 cells (*n* = 6). (D) Hepa1-6 cells were subjected to EZH2 knockdown, followed by qRT-PCR assay for the expression of miR-144 and miR-451a (*n* = 6). (E) ChIP assays were performed using antibodies of EZH2 or H3K27me3 in Hepa1-6 cell lysates to further determine the miR-144/451a promoter sites involved in EZH2 binding and H3K27me3 modification (*n* = 4). (F) The sequence of mouse chromatin region covering the miR-144/451a locus and the 5’-flanking sequences was analyzed, and the CpG islands were predicted. (G) ChIP-seq data from the Cistrome Project were used to analyze sites potentially bound by H3K27ac, H3K4me1 and H3K4me3 on the miR-144/451a locus. (H) A diagram showing the relative position of mouse miR-144/451a, the predicted CpG island and the enhancer region. (I) ChIP assays were performed using antibodies of H3K4me1, H3K4me3 and DNMT1 in lysates of AML12 and Hepa1-6 cells. Different 5’ flanking regions of the mouse miR-144/miR-451a promoter were amplified and compared (*n* = 4). (J) AML12 and Hepa1-6 cells were treated with a DNMT1 inhibitor (5-Aza) or DMSO. The expression of the indicated miRNA transcripts and targets was determined via qRT-PCR (*n* = 5). Bars, means ± SEMs; *, *P* < 0.05; **, *P* < 0.01; ***, *P* < 0.001. **Figure S22.** miR-144/451a upstream CpG island or enhancer deletion in HCC cells modulates macrophage function via paracrine factors. (A) The CpG island or the enhancer region upstream of the miR-144/451a locus was depleted in HCC cells and the expression of miR-144/miR-451a and their targets were determined via qRT-PCR (*n* = 5). (B) The supernatant of CpG island or enhancer knockout cells was harvested to measure the production of HGF and MIF via ELISA (*n* = 5). (C) The supernatant of CpG island or enhancer knockout cells was harvested and used for culture of macrophages, and the levels of polarization markers were assayed by qRT-PCR (*n* = 5). (D) The stimulated macrophages in (C) were cocultured with CFSE-stained Hepa1-6 cells. Tumor cell engulfment by macrophages was detected via FACS (n= 6). Bars, means ± SEMs; *, *P* < 0.05; **, *P* < 0.01; ***, *P* < 0.001. **Figure S23.** Blocking HGF and MIF using neutralizing antibodies relieved the effect of pri-miR-144/451a enhancer deletion in vivo. (A) The enhancer region upstream to miR-144/451a was deleted via CRISPR/cas9 system in luciferase-expressing Hepa1-6 cells, and cells were inoculated intrahepatically into C57BL/6 mice with or without combination antibodies of HGF and MIF. Tumor development was evaluated by bioluminescence imaging three weeks after the injection (*n* = 5). (B) The expression of miR-144/miR-451a in tumor tissues was measured by qRT-PCR (*n* = 5). (B) The secretion of HGF and MIF in the serum was measured via ELISA (*n* = 5). (D, E) Tumor tissues were co-stained with F4/80 and CD206 and analyzed for expression of the M2-like macrophage marker, CD206 (*n* = 5). Bars, means ± SEMs; *, *P* < 0.05; **, *P* < 0.01; ***, *P* < 0.001.

## Data Availability

The datasets used and/or analyzed during the current study are available within the manuscript and its supplementary information files.

## Abbreviations

5-Aza: 5-azacytidine
5′-RACE: Rapid amplification of 5′ cDNA ends
ΔCpG: CpG island deletion
ΔEnh: Enhancer deletion
ASOs: Anti-sense oligonucleotides
CTCF: CCCTC-binding factor
DNMTs: DNA methyltransferases
GEO: Gene Expression Omnibus
HCC: Hepatocellular carcinoma
HGF: Hepatocyte growth factor
miRNAs: microRNAs
MIF: Macrophage migration inhibitory factor
ncRNA: Noncoding RNA
NO: Nitric oxide
PRC2: Polycomb repressive complex
TAMs: Tumor-associated macrophages
TSS: Transcription start site
WT: Wild-type
