# Supplementary material for: Epigenetic silencing of miR-144/451a cluster contributes to HCC progression via paracrine HGF/MIF-mediated TAM remodeling
Source: Mol Cancer. 2021 Mar 3;20:46. doi: 10.1186/s12943-021-01343-5 (PMC7927270; doi:10.1186/s12943-021-01343-5)
Supplement: Supplementary file 1 — Additional file 1: Supplementary Materials and Methods. Table S1. Primers and oligonucleotides used in this study. Table S2. Antibodies used in this study. Figure S1. Dicer1 suppresses HCC development and correlates with a better prognosis of HCC patients. (A) The relationship between the expression of Dicer1 and the prognosis of male virus-unrelated HCC patients was evaluated using data from TCGA (n = 96). (B-D) Hepa1-6 cells after Dicer1 knockdown via vector-based shRNA transfection were used for subcutaneous inoculation of C57BL/6 mice. Mice were sacrificed four weeks after inoculation, and tumors were excised for examination (n = 5). Bars, means ± SEMs; **, P < 0.01. Figure S2. Correlation of miRNAs with HCC prognosis in published datasets. (A, B) The miRNA expression profiles in published GEO datasets (GSE140370, n = 3; and GSE128274, n = 4). (C, D) The relationship between the expression of candidate miRNAs and HCC patient prognosis was evaluated using these datasets (n = 253). Figure S3. The relationship of miR-144 and miR-451a expression in HCC (n = 125). Figure S4. The expression of miR-144 and miR-451a is repressed in human and mouse HCC cell lines and tissues. (A-D) The expression of miR-144 and miR-451a was measured via qRT-PCR in normal hepatocyte (NH) and HCC cell lines of human (A and B) and mouse (C, D) organs (n = 6). (E, F) Hepa1-6 cells were inoculated intrahepatically into C57BL/6 mice, and paratumor and HCC tissues were isolated to measure the expression of miR-144 and miR-451a (n = 5). Bars, means ± SEMs; **, P < 0.01; ***, P < 0.001. Figure S5. miR-144 or miR-451a overexpression suppresses HCC development in vivo. (A, B) Control Hepa1-6 cells or those overexpressing miR-144 or miR-451a were inoculated intrahepatically into C57BL/6 mice. Body weight and liver weight were examined three weeks after the injection (n = 6) (A). Tumors were then dissected, and the expression of miR-144/miR-451a was measured via q-RT-PCR (n = 6) (B). (C, D) H22 cells [file 12943_2021_1343_MOESM1_ESM.docx]

**Supplementary Materials**

**Supplementary Materials and Methods:**

**Cell culture**

The nucleated cells in bone marrow (BM) were harvested from the femurs and tibias of C57BL/6 mice. After erythrolysis using buffered 0.14 M NH_4_Cl, cells were cultured in Dulbecco's modified Eagle's medium (DMEM) (Gibco, Waltham, MA) containing 10% fetal calf serum (FCS) (ExCell Bio, Shanghai, China) and 2 mM L-glutamine (Gibco). For bone marrow-derived macrophage (BMDM) induction, M-CSF (25 ng/ml) (SinoBio, Beijing, China) was added to cultured cells for 7 days. Furthermore, LPS (50 ng/ml, Sigma) plus IFN-γ (20 ng/ml, SinoBio) or IL-4 (20 ng/ml, SinoBio) was used for 24 h of stimulation to obtain BMDMs with different polarizations. For in vivo experiments, TAM subgroups were sorted by a fluorescence-activated cell sorter (FACS) AriaIII flow cytometer (BD Immunocytometry Systems, Franklin Lakes, NJ) with antibodies against CD11b and F4/80. T cells were sorted from the spleens of C57BL/6 mice by magnetic beads (BD Immunocytometry Systems) with an anti-CD3 antibody. Normal hepatic cell lines of human being (QZG and HL-7702) and mice (AML12), HCC cell lines of human being (HepG2, Hep3B and Huh7) and mice (Hepa1-6, H22 and Hca-F), and human macrophage cell line THP1 and HEK293T cells were all obtained from the American Type Culture Collection (ATCC, Manassas, VA) repository from 2015 to 2019. These cells were authenticated by both morphological profiling and short tandem repeat profiling, and tested by PCR to exclude the mycoplasma contamination. All of these cells were cultured with DMEM supplemented with 10% FCS and 2 mM L-glutamine. HEK293T cells were transfected using Lipofectamine 2000^TM^ (Invitrogen, Waltham, MA) according to the manufacturer’s instructions. Where indicated, miR-144, miR-451a and shRNA for Dicer1 were delivered via recombinant lentiviruses to achieve stable expression in Hepa1-6 cells. HGF, MIF and EZH2 were also overexpressed in HCC cells by infection with lentiviruses. For the certain DNA fragment deletion, CRISPR/cas9 system was constructed by inserting sgRNA sequences targeting the miR-144/451a enhancer or CpG island into lenti-CRISPR-v2 plasmid. Then the lentiviruses were prepared and used to infect Hepa1-6 cells for depletion of the corresponding chromatin regions.

**Plasmids**

The wild-type or mutant 3’-UTRs of HGF, MIF and EZH2 were amplified by PCR with a human cDNA library as a template, and then inserted into the pGL3-promoter vector (Promega, Fitchburg, WI) to construct reporter plasmids. Moreover, the different truncated fragments of the pri-miR-144/451a promoter were amplified by PCR with mouse genomic DNA as a template. These fragments were inserted into the pGL3-promoter vector to construct reporters. Full-length HGF, MIF and EZH2 ORFs were amplified from a human cDNA library and inserted into pFlag-CMV2 (Invitrogen) to construct overexpression plasmids. The packaging of lentiviruses overexpressing miR-144, miR-451a, HGF, MIF and EZH2 was performed by GeneChem Company (GeneChem, Shanghai, China).

**Tumor-bearing mouse model**

Hepa1-6 cells were used to establish orthotopic HCC models in mice. Briefly, C57BL/6 mice were anesthetized by intraperitoneal (i.p.) injection of 0.6% pentobarbital sodium (10 µl/g, Sigma-Aldrich, St. Louis, MO). Hepa1-6 cells were infected with lentiviruses expressing firefly luciferase (GeneChem, Shanghai, China) following the manufacturer’s protocol and then injected (5 × 10^6^ cells in 30 μl Matrigel, Sigma-Aldrich) into the liver parenchyma of the left lobe. Hepatic tumor growth was monitored using an in vivo imaging system (IVIS) (Xenogen, Perkin-Elmer, Fremont, CA). Mice were sacrificed 3 weeks after the injection. Tumors were weighed, and tumor volume was estimated as (L × S^2^) × 0.51 (L, long diameter; S, short diameter). Paratumor or tumor tissues were obtained and preserved in 4% paraformaldehyde for pathological analysis, and the rest of the tumor was minced and digested into a single cell suspension with type V collagenase and DNase incubation for 40 min for further analysis. Hepa1-6 cells (5 × 10^6^ cells) infected by lentiviruses expressing Dicer1-targeted shRNAs or H22 cells (5 × 10^6^ cells) infected by lentiviruses expressing miR-144/miR-451a were injected subcutaneously into the right back of recipient mice.

**CCK-8 assay**

The proliferation of Hepa1-6 cells was analyzed with CCK8 assay. Briefly, control Hepa1-6 cells or those overexpressing miR-144/miR-451a were seeded into 96-well plates, and assayed at 24 h, 48 h, 72 h and 96 h using the CCK8 reagent kit (7sea Biotech, Shanghai, China). After incubation for 4h at 37°C, the supernatant was removed and the precipitant was dissolved in DMSO (Sigma). Spectrophotometric absorbance was measured at the wavelength of 490 nm by a microplate reader (BioTek Instruments Inc., Winooski, VT).

**ELISA**

According to the manufacturer’s instructions, cytokines concentration was determined using the ELISA Kits (Abcam, Cambridge, MA). Briefly, after different treatment, the supernatant of BMDMs was treated with antibody cocktail and incubated with shaking for 1 h. Then, 100 µL of substrate was added to samples and incubated with shaking for 10 min in the dark. Finally, 100 µL of Stop Solution was added to the samples. The absorption at 450 nm was recorded using a microplate reader (Thermo, Waltham, MA).

**Reverse transcription-polymerase chain reaction (RT-PCR)**

Total RNA was prepared from cells using TRIzol reagent (Invitrogen) according to the manufacturer’s instructions. cDNA was synthesized using a PrimeScript RT Reagent Kit (TaKaRa BIO, Dalian, China). Real-time PCR was performed using a SYBR Premix Ex Taq Kit (TaKaRa) and an ABI PRISM 7500 Real-time PCR system (Life Technologies, Waltham, MA), with β-actin or U6 RNA (for miRNAs) as internal controls. Rapid amplification of cDNA ends (RACE) was performed using a SMARTer RACE cDNA Amplification Kit (Clontech, Shiga, Japan). PCR primers are shown in Table S1.

**Flow cytometry and immunofluorescence**

Cells were stained with different antibodies listed in Table S2. FACS analysis was performed with routine protocols using a FACS CantoPlus flow cytometer (BD Immunocytometry Systems). Data were analyzed with FlowJo vX.0.6 software (FlowJo, LLC, Ashland, OR). Dead cells were identified by 7-AAD staining and excluded. Apoptosis of Hepa1-6 cells was examined by a Dead Cell Apoptosis Kit with Annexin V/PI (eBioscience). For cell cycle assays, Hepa1-6 cells were collected and fixed in cold 70% ethanol (V/V) overnight at 4 °C. Cells were stained with 50 μg/ml propidium iodide (Sigma) in the dark, followed by incubation with 20 g/ml RNase A (Sigma) for 30 min at 37 °C. The DNA content was analyzed by flow cytometry. HCC tissues were stained with F4/80 (eBioscience), CD206 (Abcam), p65 (Abcam), Ki67 (Lab Vision, Milpitas, CA) or CD31 (Biolegend, San Diego, CA) and observed under a laser scanning confocal microscope (FV-1000, Olympus, Tokyo, Japan).

**Western blot**

Whole-cell lysates were prepared with RIPA buffer (Beyotime, Haimen, China). Protein concentration was determined with a BCA Protein Assay Kit (Pierce, Waltham, MA). The samples were separated by SDS-PAGE, blotted onto polyvinylidene fluoride (PVDF) membranes and probed with primary antibodies listed in Table S2, followed by an incubation with horseradish peroxidase (HRP)-conjugated goat anti-rabbit IgG or goat anti-mouse IgG (Boster Bio Tec, Wuhan, China). Membranes were visualized with an ECL detection system (Pierce).

**Reporter assay**

Hepa1-6 cells were transfected with different reporter combinations of the 3’-UTR (miRNA targets) or promoter region, expression vectors (miRNA or EZH2) and Renilla luciferase vectors (phRL-TK, Promega) using Lipofectamine 2000^TM^. Cells were harvested and lysed 48 h after transfection, and the relative luciferase activity was read with a Dual-Luciferase Reporter Assay System (Gloma X^TM^ 20/20 Luminometer, Promega).

**Chromatin immunoprecipitation (ChIP)**

ChIP assays were performed using a kit (Merck Millipore, Billerica, MA) according to the manufacturer’s instructions. Briefly, cells were fixed in 1% formaldehyde, and crosslinked protein-DNA complexes were fragmented into 200 ~ 500 bp fragments by ultrasonic sound waves. Immunoprecipitation was performed using the antibodies shown in Table S2. Immune complexes were collected using protein G-Sepharose beads, washed, eluted and incubated for 5 h at 65 °C to reverse the cross-linking. DNA was extracted and analyzed by PCR using the primers listed in Table S1.

**NO production assays**

BMDMs were cultured, and 50 μl culture supernatants were added to 50 μl Griess reagent I and 50 μl Griess reagent II (Beyotime). Absorbance was measured at 540 nm with a microplate reader.

**Tumor cell phagocytosis and cytotoxicity experiments**

Hepa1-6 cells were incubated with carboxyfluorescein succinimidyl amino ester (CFSE) (5 nM; Dojindo Molecular Technologies, Inc., Kumamoto, Japan) at room temperature for 15 min, followed by washing with PBS. BMDMs (2 × 10^5^ cells) with different treatments were cocultured with CFSE-stained Hepa1-6 cells (6 × 10^5^ cells) in 12-well plates for incubation at 37 °C for 2 h. The samples were stained with F4/80 (BM8, eBioscience), and F4/80^+^CFSE^+^ cells represented macrophages that had engulfed Hepa1-6 cells. FACS was performed to detect phagocytosis efficiency.

For tumor cell killing assay, Hepa1-6 cells (5 × 10^6^) were repeatedly frozen to prepare the crude Hepa1-6 tumor antigen. The stimulated BMDMs were treated with supernatant of different HCC cells and incubated with thawed cell debris of Hepa1-6 for 24 h. Macrophages (2 × 10^5^) were irradiated and incubated with T cells (1 × 10^6^) sorted from lymphoid node in 96-well plates for 3 days to stimulate T cells activation. Hepa1-6 cells were then added into the culture in ratios of 1:10, 1:20, 1:40, and 1:80 (target cells : T cells). The cytotoxicity against Hepa1-6 cells was assessed using a lactate dehydrogenase (LDH) activity determination kit (Cayman Chemical Company, Ann Arbor, MI).

**Chromosome conformation capture (3C)**

Normal hepatic and HCC cells with different treatments were prepared and crosslinked with 1% formaldehyde to maintain the three-dimensional conformation of chromatin. Then, the chromatin was sheared by the restriction endonuclease HindIII to separate the interacting DNA regions from other noninteracting DNA. DNA fragments that interacted in the same complex were ligated to each other by the cohesive ends to form a loop, followed by incubation for 5 h at 65 °C to reverse the cross-linking. The interaction between the regulatory elements was validated using PCR with the primers shown in Table S1.

**Bisulfite sequencing PCR (BSP)**

Genomic DNA was extracted from paratumor and HCC tissues, and then 4 μg of DNA was incubated with 3.6 M bisulfite overnight at 50 °C to convert unmethylated cytosine to uracil. Then, the modified DNA was purified by a Cleanup DNA purification Kit (Promega, Fitchburg, WI) according to the protocol. Then, the predicted CpG island was amplified via PCR with primers in Table S1, followed by Sanger sequencing to analyze the methylation of CpG dinucleotides.

**Cytokine antibody array**

The sera of 36 HCC patients and supernatants of control HCC cells and those overexpressing miR-144 or miR-451a were prepared. The abundances of cancer-secreted cytokines were determined using the MILLIPLEX MAP Kit – Cancer Biomarker Panel (Millipore, Billerica, MA, USA) based on the Luminex technology on the Bio-Plex 200 System (Bio-Rad Laboratories, Hercules, Ca).

**Supplementary Tables:**

**Table S1. Primers and oligonucleotides used in this study**

| **Name** | **Purpose** | **Sequence** |
| --- | --- | --- |
| mIL-12-F  mIL-12-R  mTNF-α-F  mTNF-α-R  mIL-10-F  mIL-10-R  mTGF-β-F  mTGF-β-R  mHGF-F  mHGF-R  mMIF-F  mMIF-R  mEZH2-F  mEZH2-R  mβ-actin-F  mβ-actin-R  miNOS-F  miNOS-R  mMR-F  mMR-R  hCD68-F  hCD68-R  hCD163-F  hCD163-R  hHLA-DR-F  hHLA-DR-R  hHGF-F  hHGF-R  hMIF-F  hMIF-R  hEZH2-F  hEZH2-R  hIL-12-F  hIL-12-R  hIL-10-F  hIL-10-R  TSS -300bp-F  TSS -300bp-R  TSS -200bp-F  TSS -200bp-R  TSS -100bp-F  TSS -100bp-R  Pro -4.5kb-F  Pro -4.5kb-R  Pro -3.5kb-F  Pro -3.5kb-R  Pro -3.0kb-F  Pro -3.0kb-R  Pro -1.5kb-F  Pro -1.5kb-R  Pro -0.5kb-F  Pro -0.5kb-R  Pro +0.5kb-F  Pro +0.5kb-R  Pro +1.5kb-F  Pro +1.5kb-R  P  P’  P1  P2  P3  P4  P5  P6  P7  Dicer1 shRNA  EZH2 shRNA | RT-PCR  RT-PCR  RT-PCR  RT-PCR  RT-PCR  RT-PCR  RT-PCR  RT-PCR  RT-PCR  RT-PCR  RT-PCR  RT-PCR  RT-PCR  RT-PCR  RT-PCR  RT-PCR  RT-PCR  RT-PCR  RT-PCR  RT-PCR  RT-PCR  RT-PCR  RT-PCR  RT-PCR  RT-PCR  RT-PCR  RT-PCR  RT-PCR  RT-PCR  RT-PCR  RT-PCR  RT-PCR  RT-PCR  RT-PCR  RT-PCR  RT-PCR  ChIP  ChIP  ChIP  ChIP  ChIP  ChIP  ChIP  ChIP  ChIP  ChIP  ChIP  ChIP  ChIP  ChIP  ChIP  ChIP  ChIP  ChIP  ChIP  ChIP  3C  3C  3C  3C  3C  3C  3C  3C  3C  KD  KD | 5’-GGAAGCACGGCAGCAGAATA  5’-AACTTGAGGGAGAAGTAGGAATGG  5’-CAGGAGGGAGAACAGAAACTCCA  5’-CCTGGTTGGCTGCTTGCTT  5’-CCCTTTGCTATGGTGTCCTT  5’-TGGTTTCTCTTCCCAAGACC  5’-GACCGCAACAACGCCATCTA  5’-GGCGTATCAGTGGGGGTCAG  5’-ATGTGGGGGACCAAACTTCTG  5’-GGATGGCGACATGAAGCAG  5’-GCCAGAGGGGTTTCTGTCG  5’-GTTCGTGCCGCTAAAAGTCA  5’-TGCCTCCTGAATGTACTCCAA  5’-AGGGATGTAGGAAGCAGTCATAC  5’-GGCTGTATTCCCCTCCATCG  5’-CCAGTTGGTAACAATGCCATGT  5’-GTTCTCAGCCCAACAATACAAGA  5’-GTGGACGGGTCGATGTCAC  5’-CTCTGTTCAGCTATTGGACGC  5’-CGGAATTTCTGGGATTCAGCTTC  5’-GGAAATGCCACGGTTCATCCA  5’-TGGGGTTCAGTACAGAGATGC  5’-TTTGTCAACTTGAGTCCCTTCAC  5’-TCCCGCTACACTTGTTTTCAC  5’-AGTCCCTGTGCTAGGATTTTTCA  5’-ACATAAACTCGCCTGATTGGTC  5’-GCTATCGGGGTAAAGACCTACA  5’-CGTAGCGTACCTCTGGATTGC  5’-TCCTACAGCAAGCTGCTGT  5’-CTGCGGCTCTTAGGCGAA  5’-AATCAGAGTACATGCGACTGAGA  5’-GCTGTATCCTTCGCTGTTTCC  5’-ACCCTGACCATCCAAGTCAAA  5’-TTGGCCTCGCATCTTAGAAAG  5’-GACTTTAAGGGTTACCTGGGTTG  5’-TCACATGCGCCTTGATGTCTG  5’-GGCAAAGAACCAAACAGTGCT  5’-AGAGAGACTCTGCCTAGCACT  5’-CTGGGAATAGAAATTTAGGCT  5’-TAGGCAATGTTAGTTGAATCT  5’-CTGACCAGCAATTAAATGCT  5’-TAGAAGGCCTTTTCTCACAT  5’-TAAGAGTAATTCTACCCGCT  5’-TGGTGGCACATGCCTGTATT  5’-AAGGCCCTAGCTGGTGTCCT  5’-CCAGGGCTGTTTTCCTGGAT  5’-AAGGAGCTTGAGCTGTCTGT  5’-GATGAGCGACAAAGCTTCT  5’-ATAGAAGTACATCAGTGAGCT  5’-AGTAGAGACAGGCTTTCACCGT  5’-ACCTCAGGAAATAGAACACCT  5’-AGGCCTCTGCTGTTTAGAAT  5’-CCCTGACCTGTCCTGTTCT  5’-TTGTCAGGCTCCAGAGCT  5’-TACACCTTTGTAGCTGTGGT  5’-TGAACACTGACTGGCCACT  5’-AATATGGTCCATGAGAGAGT  5’-ACTCTCTCATGGACCATATT  5’-CTTCATCCTTTGTAGCCT  5’-CAGTACCTACTTACCAGCT  5’-ACCTGGGACCATTGCAAGAA  5’-ACAGACAGCTCAAGCTCCTT  5’-AGAACAATGCATGACTCAGA  5’-ACAGAGTGAAACTCCATC  5’-AGCTGCAATCCAGCAACA  5’-GCGCAAATACAAGCCCTATGA  5’-GCACAAGTCATCCCGTTAAAG |

*F, forward; R, reverse.

**Table S2. Antibodies used in this study**

| **Antigen** | **Clone#** | **Supplier** |
| --- | --- | --- |
| CD68  F4/80  CD206  P65  Arg1  HGF  MIF  EZH2  H3K27me3  H3K4me1  H3K4me3  DNMT1  β-actin  CD31  APC-CD11b  Alexa488-F4/80  Biotin-Ly6G  APC-Ly6C  Marco  CD86  MHCII  CD11c  BV421-CD45  PE-CD4  APC-CD8  FITC-CD3  FOXP3  CD25  APC-B220  Biotin-NK1.1 | 514H12  BM8  15-2  E379  D4E3M  198811  932612  EPR20108  mAbcam 6002  polyclonal  polyclonal  60B1220.1  AC-15  MEC13.3  M1/70  BM8  1A8  HK1.4  PLK-1  A17199A  KH74  N418  30-F11  RM4-5  53-6.7  17A2  206D  BC96  RA3-6B2  PK136 | Thermo Fisher  eBioscience  Abcam  Abcam  Cell Signaling Technology  R&D Systems  R&D Systems  Abcam  Abcam  Abcam  Abcam  Abcam  Sigma  BD  eBioscience  eBioscience  eBioscience  eBioscience  eBioscience  Biolegend  BD  Biolegend  BD  BD  BD  BD  Biolegend  Biolegend  Biolegend  Biolegend |

**Supplementary Figures:**


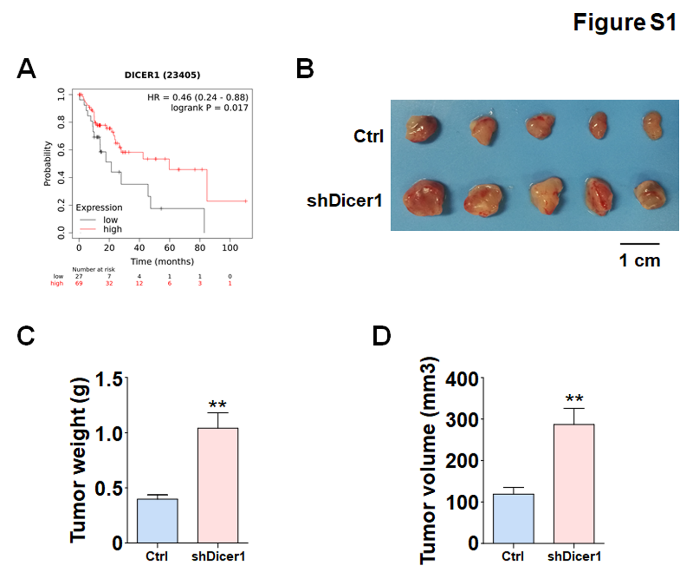


**Figure S1. Dicer1 suppresses HCC development and correlates with a better prognosis of HCC patients.** (A) The relationship between the expression of Dicer1 and the prognosis of male virus-unrelated HCC patients was evaluated using data from TCGA (n = 96). (B-D) Hepa1-6 cells after Dicer1 knockdown via vector-based shRNA transfection were used for subcutaneous inoculation of C57BL/6 mice. Mice were sacrificed four weeks after inoculation, and tumors were excised for examination (n = 5). Bars, means ± SEMs; **, P < 0.01.


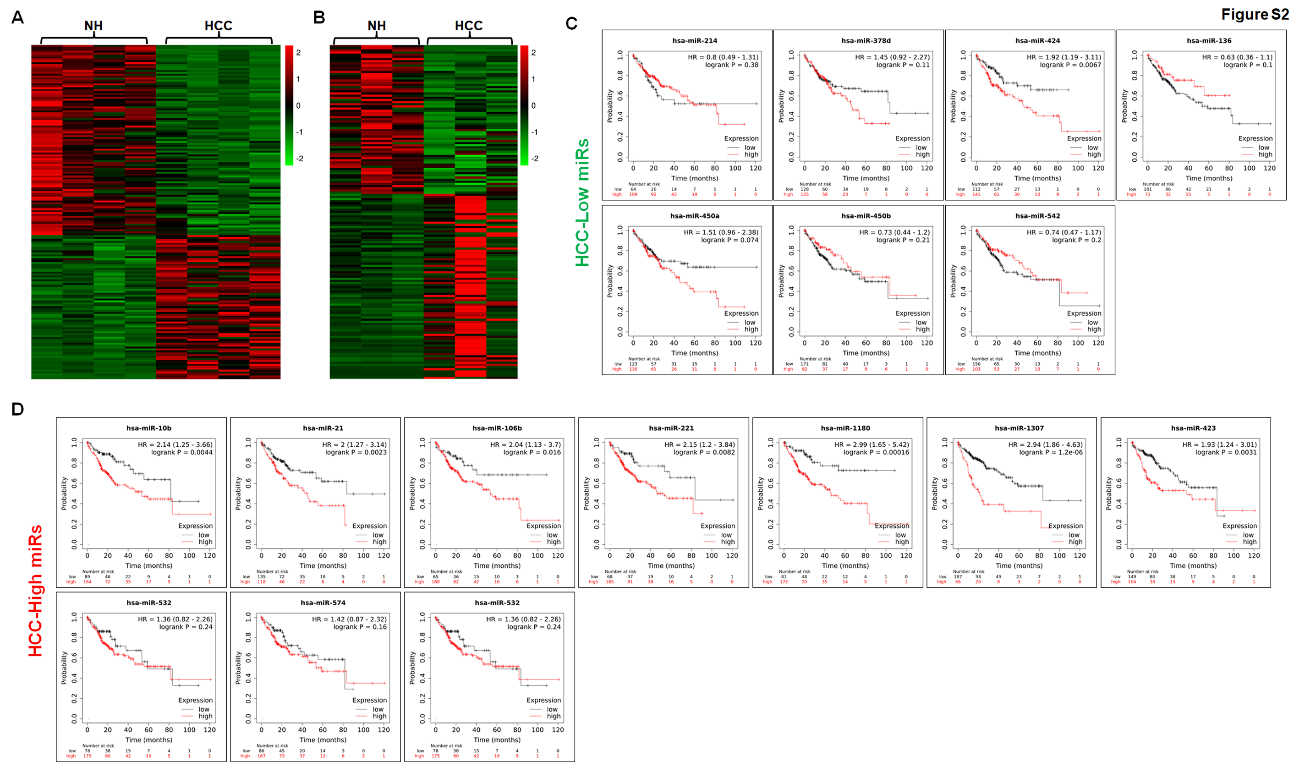


**Figure S2. Correlation of miRNAs with HCC prognosis in published datasets.** (A, B) The miRNA expression profiles in published GEO datasets (GSE140370, n = 3; and GSE128274, n = 4). (C, D) The relationship between the expression of candidate miRNAs and HCC patient prognosis was evaluated using these datasets (n = 253).


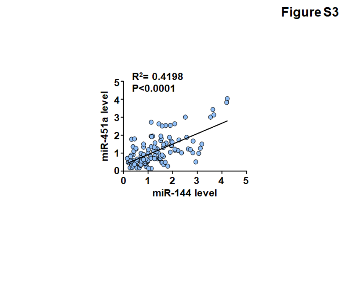


**Figure S3.** **The relationship of miR-144 and miR-451a expression in HCC (n = 125).**


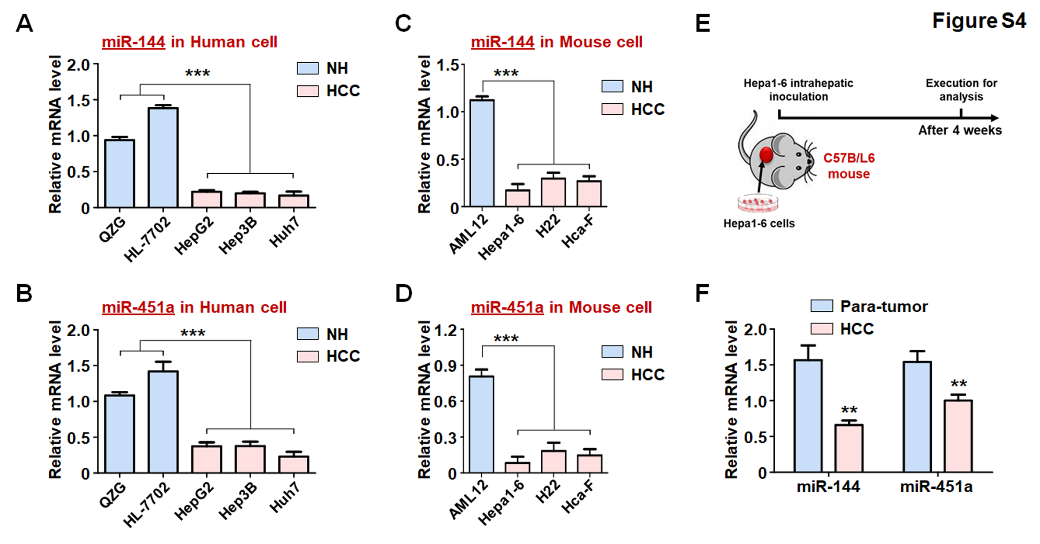


**Figure S4. The expression of miR-144 and miR-451a is repressed in human and mouse HCC cell lines and tissues.** (A-D) The expression of miR-144 and miR-451a was measured via qRT-PCR in normal hepatocyte (NH) and HCC cell lines of human (A and B) and mouse (C, D) organs (n = 6). (E, F) Hepa1-6 cells were inoculated intrahepatically into C57BL/6 mice, and paratumor and HCC tissues were isolated to measure the expression of miR-144 and miR-451a (n = 5). Bars, means ± SEMs; **, P < 0.01; ***, P < 0.001.


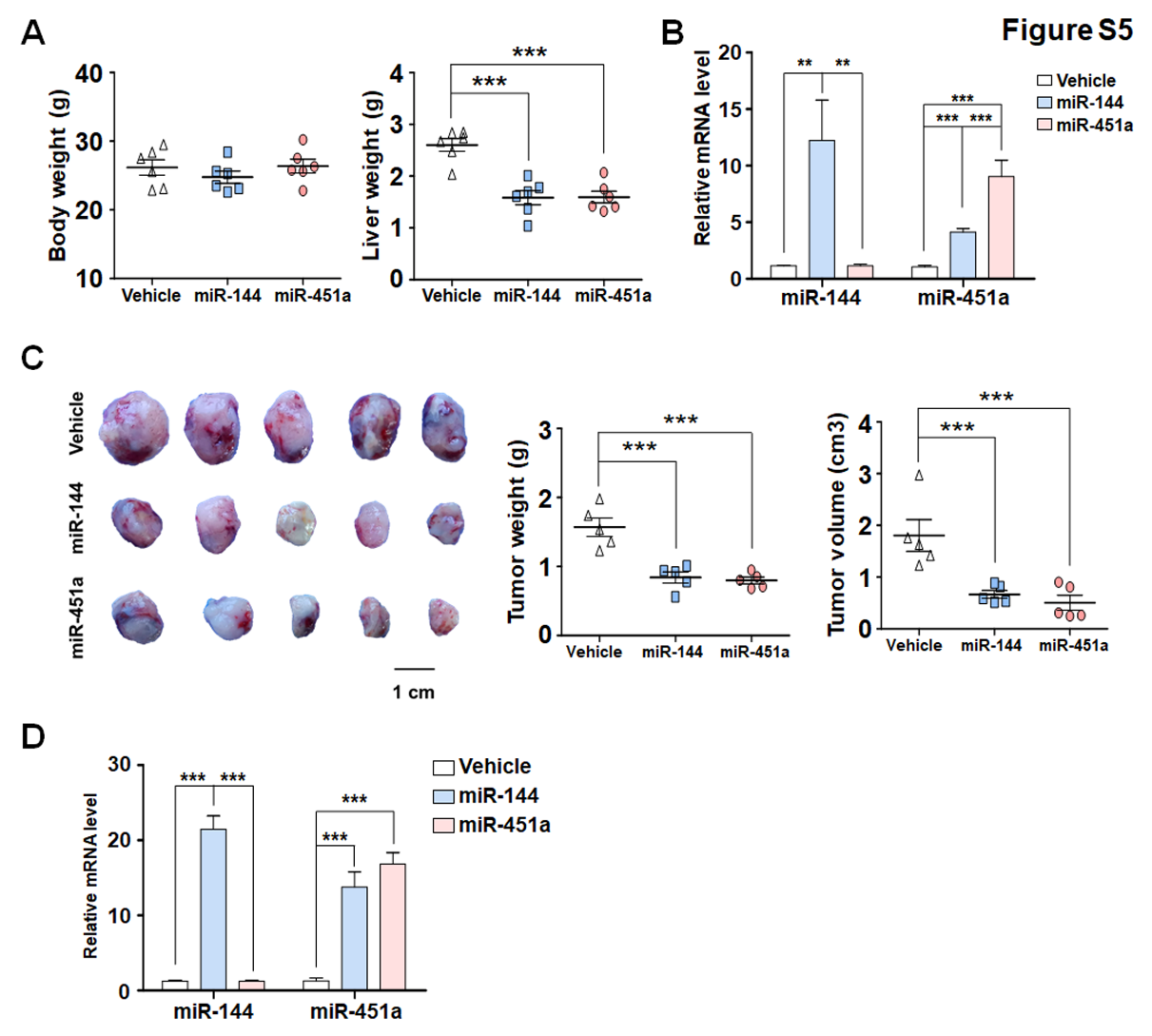


**Figure S5. miR-144 or miR-451a overexpression suppresses HCC development in vivo.** (A, B) Control Hepa1-6 cells or those overexpressing miR-144 or miR-451a were inoculated intrahepatically into C57BL/6 mice. Body weight and liver weight were examined three weeks after the injection (n = 6) (A). Tumors were then dissected, and the expression of miR-144/miR-451a was measured via q-RT-PCR (n = 6) (B). (C, D) H22 cells were infected with control or miR-144/miR-451a-overexpressing lentiviruses and were subcutaneously inoculated on C57BL/6 mice. Tumor tissues were excised and weighed three weeks after the inoculation (n = 5). The expression of miR-144/miR-451a was measured via q-RT-PCR (n = 5) (D). Bars, means ± SEMs; **, P < 0.01; ***, P < 0.001.


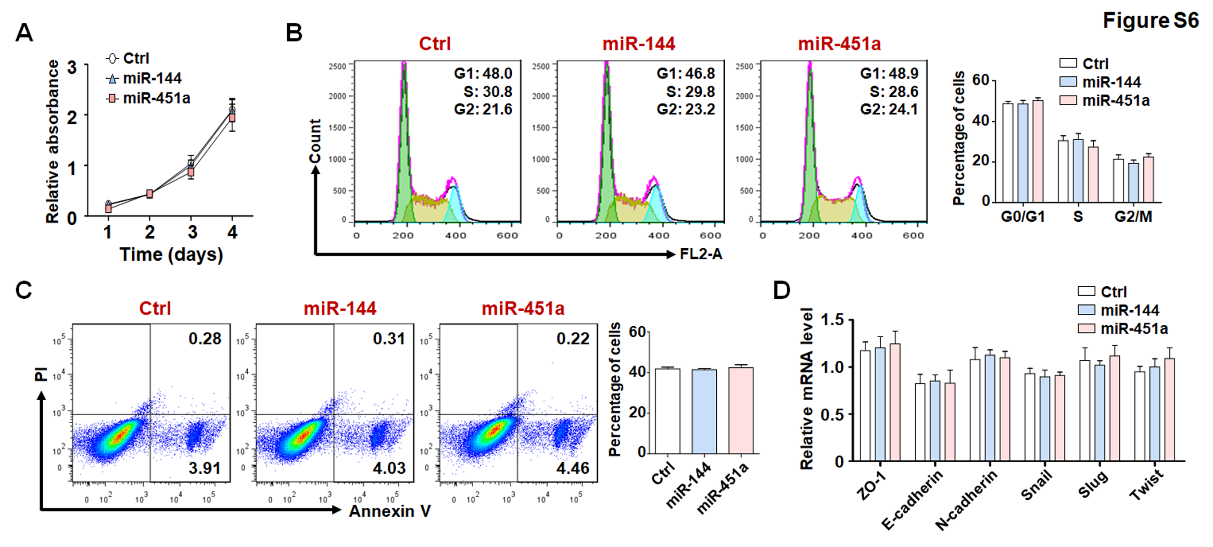


**Figure S6. miR-144 or miR-451a had no effect on proliferation and apoptosis of HCC cells.** Hepa1-6 cells were infected by control or miR-144/miR-451a-overexpressing lentiviruses. Cells were then subject to CCK-8 assay (A, n = 5), FACS assay for cell cycle pregression (B, n = 5) and apoptosis (C, n = 5). The expressions of epithelial and mesenchymal markers were also examined via qRT-PCR (D, n = 5). Bars, means ± SEM.


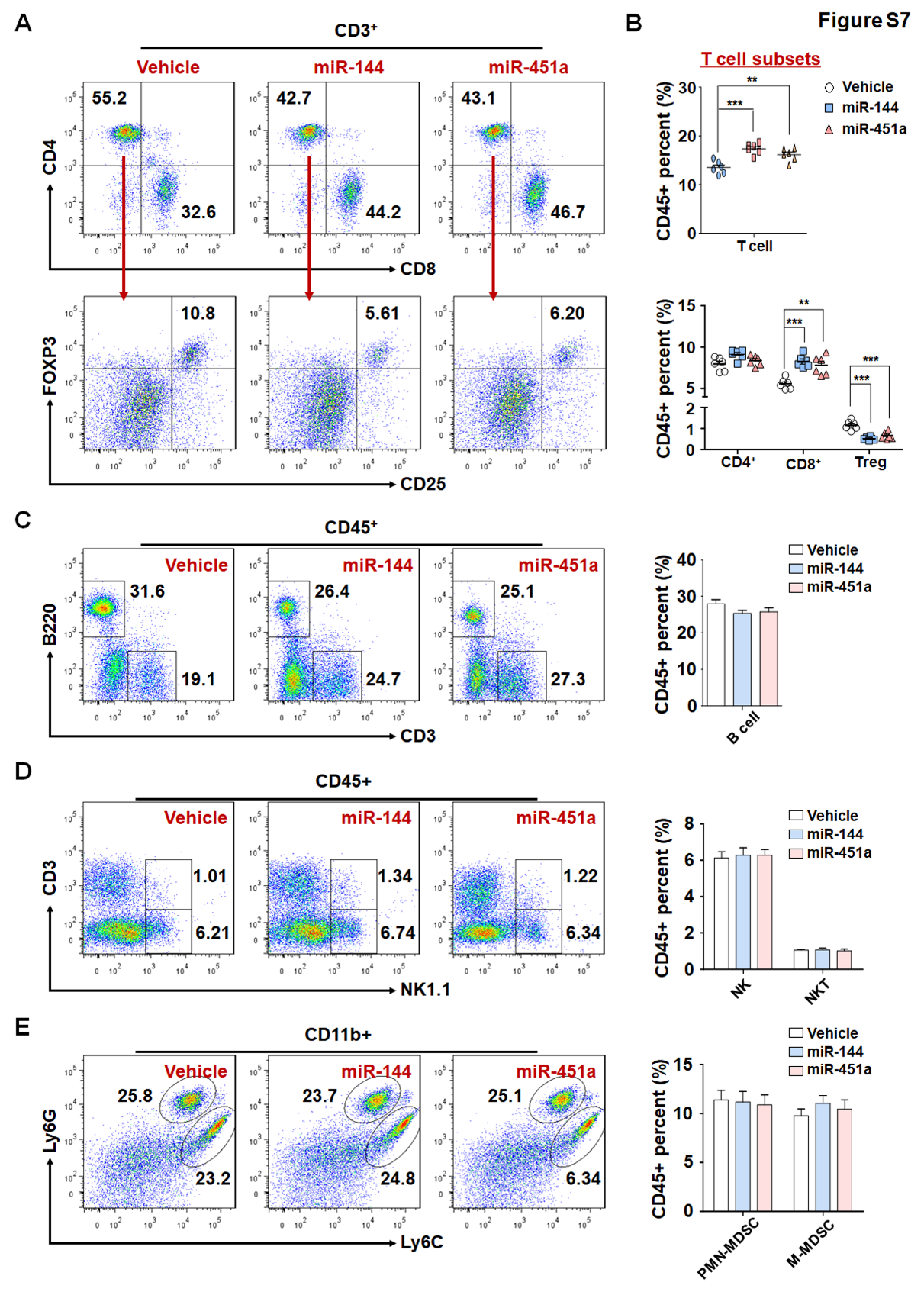


**Figure S7. miR-144/miR-451a overexpression affects microenvironmental cell subsets in an orthotopic HCC model.** (A-E) Control Hepa1-6 cells or those overexpressing miR-144 or miR-451a were inoculated intrahepatically into C57BL/6 mice. Tumors were then dissected, and FACS was performed to analyze the subsets of lymphocytes (A-D) and MDSCs (E) (n = 6). Bars, means ± SEMs; **, P < 0.01; ***, P < 0.001.


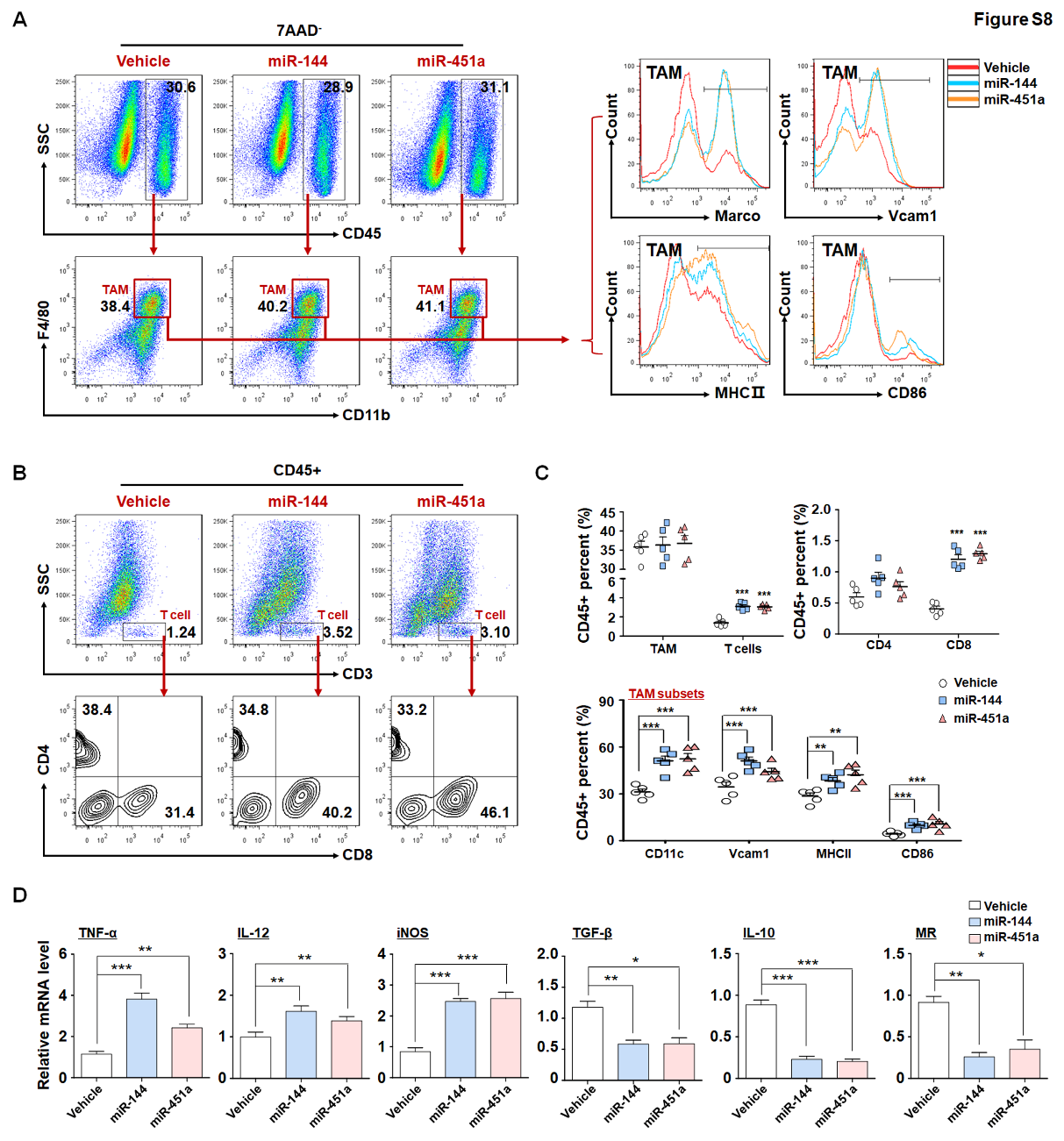


**Figure S8. miR-144/miR-451a overexpression modulates the phenotypes of microenvironmental cells in an orthotopic HCC model derived from H22 cells.**

(A-D) H22 cells were infected with control or miR-144/miR-451a-overexpressing lentiviruses and were subcutaneously inoculated on C57BL/6 mice. The tumor tissues were excised, and FACS was performed to analyze the subsets and differentiation of TAMs (A) and phenotype of lymphocytes (B) from different groups, and the cell percentages were plotted and compared (C) (n = 5). The mRNA levels of macrophage polarization markers were detected using qRT-PCR in sorted TAMs (D, n = 5). Bars, means ± SEMs; *, P < 0.05; **, P < 0.01; ***, P < 0.001.


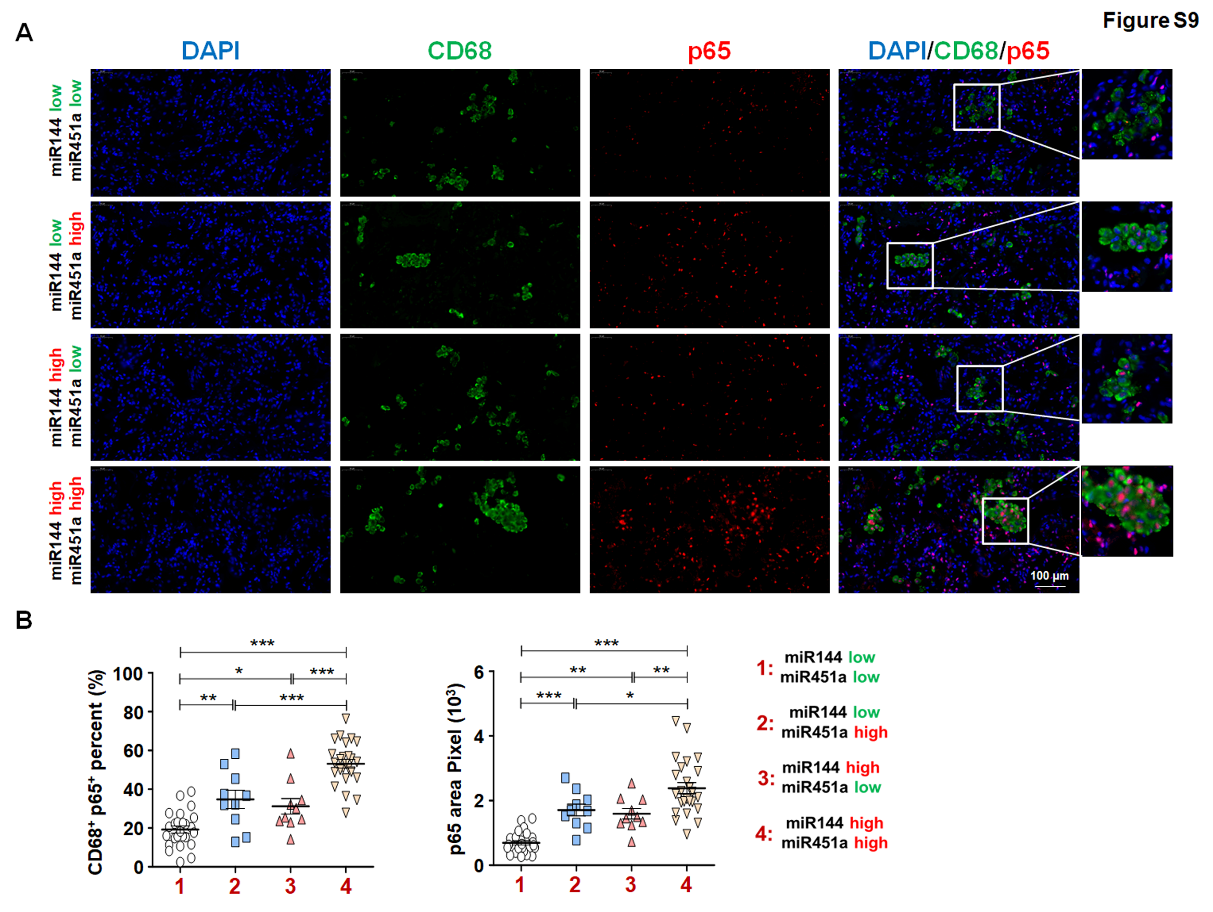


**Figure S9. The miR-144/miR-451a cluster correlates with M1-polarization of microenvironmental macrophages.** (A, B) The tumor tissues of HCC patients were stained with F4/80 and p65 (A) and analyzed for expression of indicated markers (B) (1: n = 25; 2: n = 10; 3: n = 10; 4: n = 25).


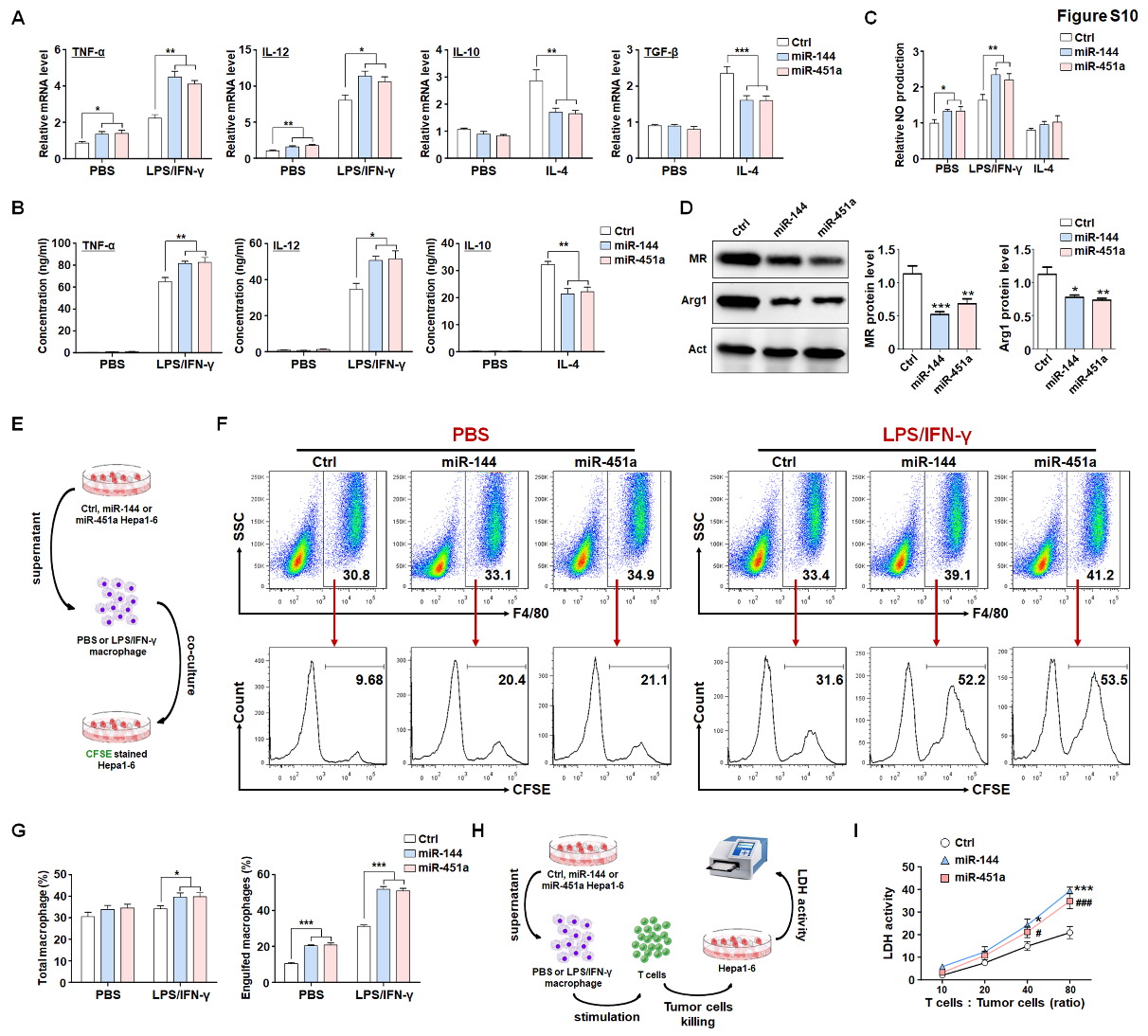


**Figure S10. miR-144/miR-451a modulates the HCC paracrine function to promote macrophage M1-like polarization and antitumor effects *in vitro*.** (A-D) BMDMs were cultured from bone marrow cells via M-CSF stimulation. Hepa1-6 cells were infected by recombinant lentiviruses to overexpress miR-144 or miR-451a, and the supernatant was prepared to culture BMDMs with the indicated stimuli. The function of macrophages was determined by qRT-PCR (A, n = 6), ELISA (B, n = 6) and Western blot (D, n =4) assays for the expression of the indicated genes or measurement of NO generation (C, n = 8). (E-G) BMDMs were incubated in the supernatant of Hepa1-6 cells as described in (A) and then cocultured with CFSE-stained Hepa1-6 cells (E). Differentiated macrophages were identified by staining for F4/80, and tumor cell engulfment by macrophages was detected via FACS (F, G) (n= 6). (H, I) BMDMs were incubated with the supernatant of Hepa1-6 cells as described in (A) and then cultured for 3 days with CD3+ T cells sorted from the spleen by magnetic beads. The activated T cells were cocultured with Hepa1-6 cells at different ratios of cell numbers. The cytotoxicity of tumor cells was assessed by LDH activity determination (n = 6). Bars, means ± SEMs; *, P < 0.05; **, P < 0.01; ***, P < 0.001.


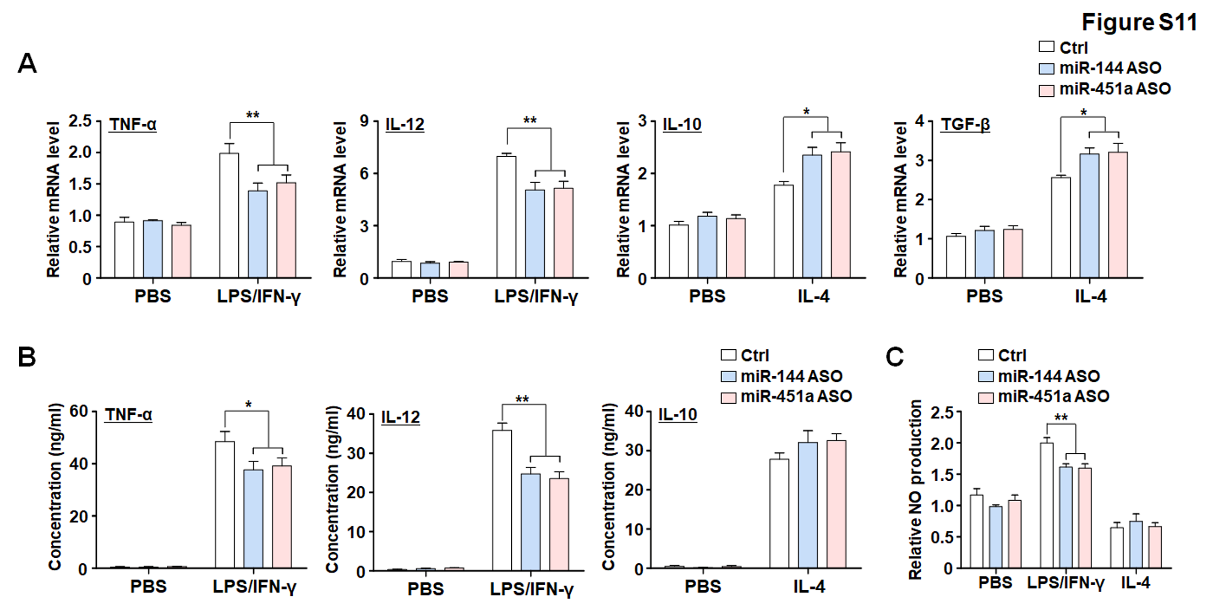


**Figure S11. Inhibition of miR-144 or miR-451a in HCC cells represses macrophage M1-like polarization via the paracrine pathway.** BMDMs were cultured from bone marrow cells via M-CSF stimulation. Hepa1-6 cells were transfected with ASOs of miR-144 or miR-451a, and the supernatant was collected for culturing BMDMs, which were further subjected to polarization stimulation with the indicated factors. The function of macrophages was determined by measuring the expression of cytokines via qRT-PCR (A), production of these cytokines via ELISA (B) and generation of NO (C) (n = 5). Bars, means ± SEMs; *, P < 0.05; **, P < 0.01.


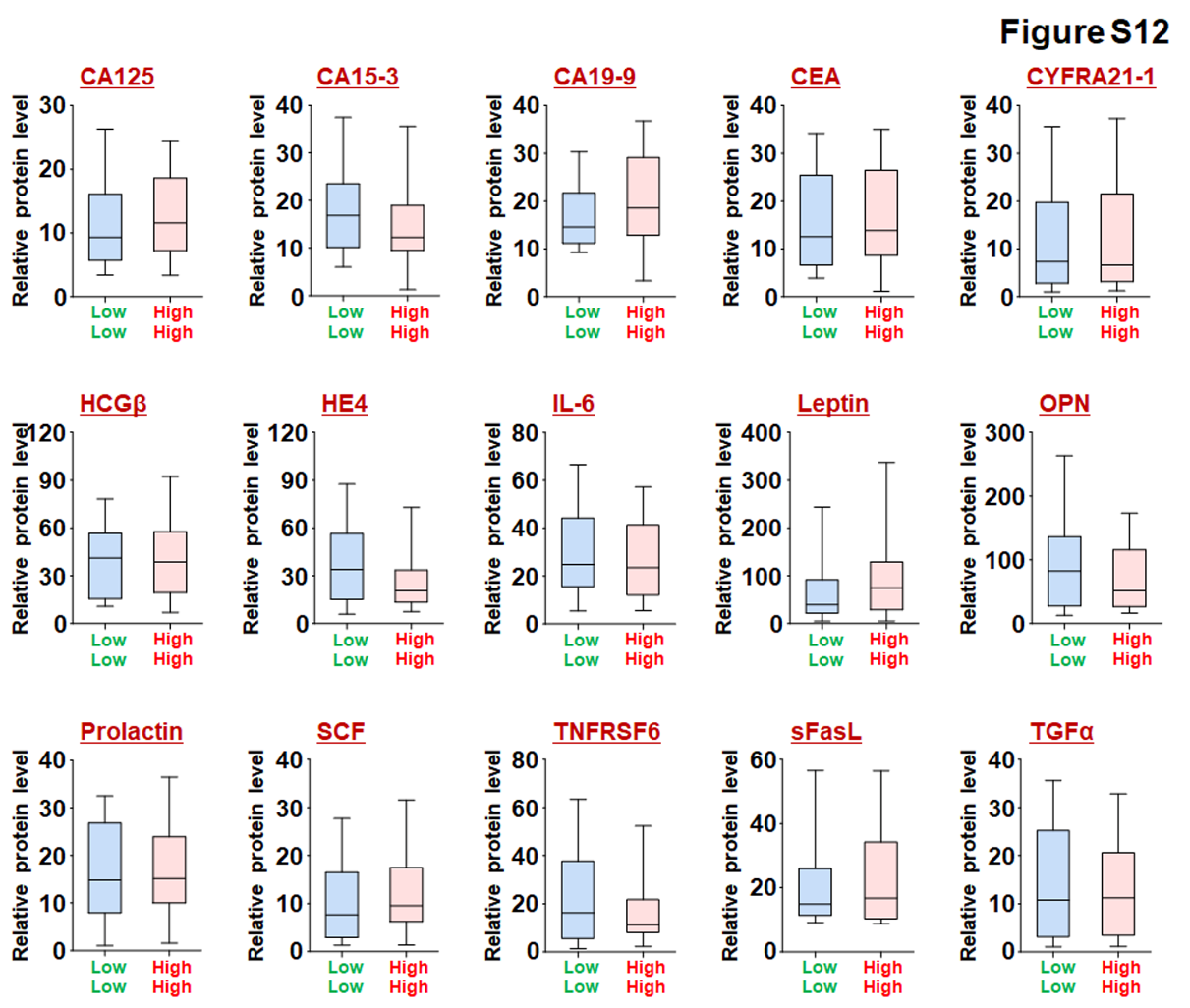


**Figure S12. The abundances of the cancer secreted cytokines were determined by cytokine antibody array from serum of HCC patients (n = 36).** Bars, means ± SEMs.


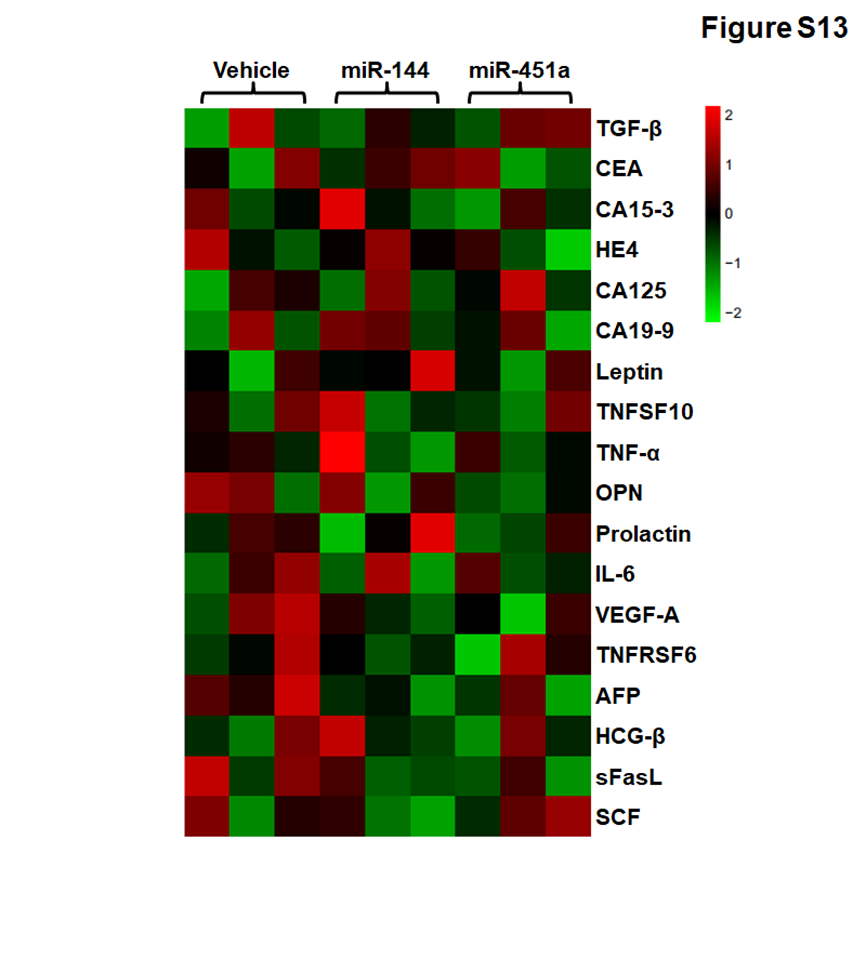


**Figure S13. The abundances of the cancer secreted cytokines which showed no difference among supernatant of Hepa1-6 cells with different treatment by cytokine antibody array (n = 3).**


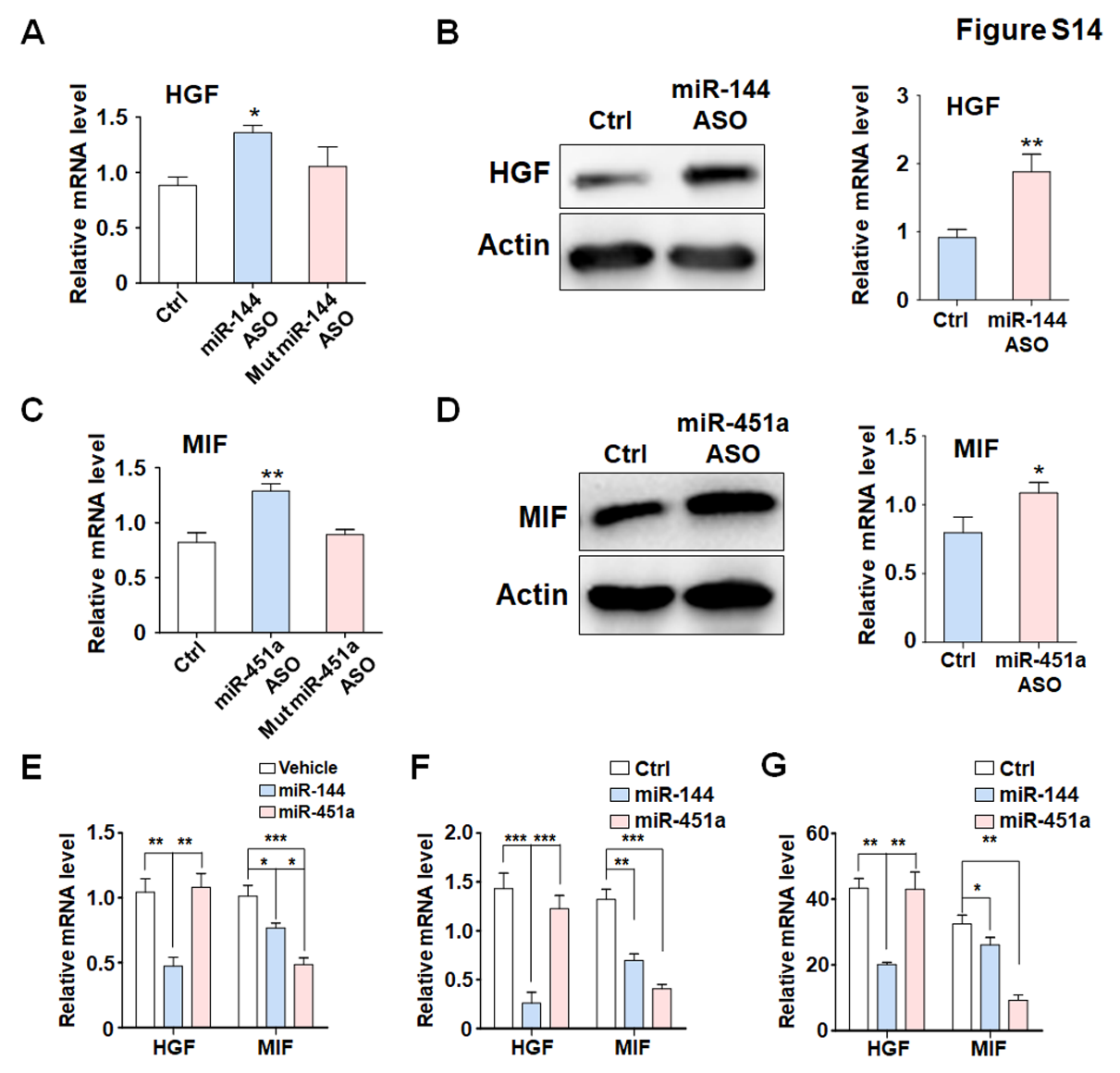


**Figure S14. miR-144 and miR-451a inhibit the expression of HGF and MIF, respectively, both in vitro and in vivo.** (A, B) Hepa1-6 cells were subjected to ASO-mediated inhibition of miR-144 or seed sequence mutated miR-144 (Mut-miR-144), followed by qRT-PCR (A) and Western blot (B) analyses for the expression of HGF (n = 6). (C, D) Hepa1-6 cells were subjected to ASO-mediated inhibition of miR-451a or seed sequence mutated miR-451a (Mut-miR-451a) followed by qRT-PCR (C) and Western blot (D) analyses for the expression of MIF (n = 6). (E) Hepa1-6 cells were infected with control or miR-144/miR-451a lentiviruses and inoculated intrahepatically into C57BL/6 mice. The tumor tissues were excised three weeks after the inoculation, and the expression of HGF and MIF was examined viaqRT-PCR. (F, G) H22 cells were infected with control or miR-144/miR-451a-overexpressing lentiviruses and were subcutaneously inoculated on C57BL/6 mice. Tumor tissues were excised and the serum was collected three weeks after inoculation. HGF and MIF mRNA levels in tumor tissues (F) and protein levels in the serum (G) were examined via qRT-PCR and ELISA, respectively (n = 5). Bars, means ± SEMs; *, P < 0.05; **, P < 0.01; ***, P < 0.001.


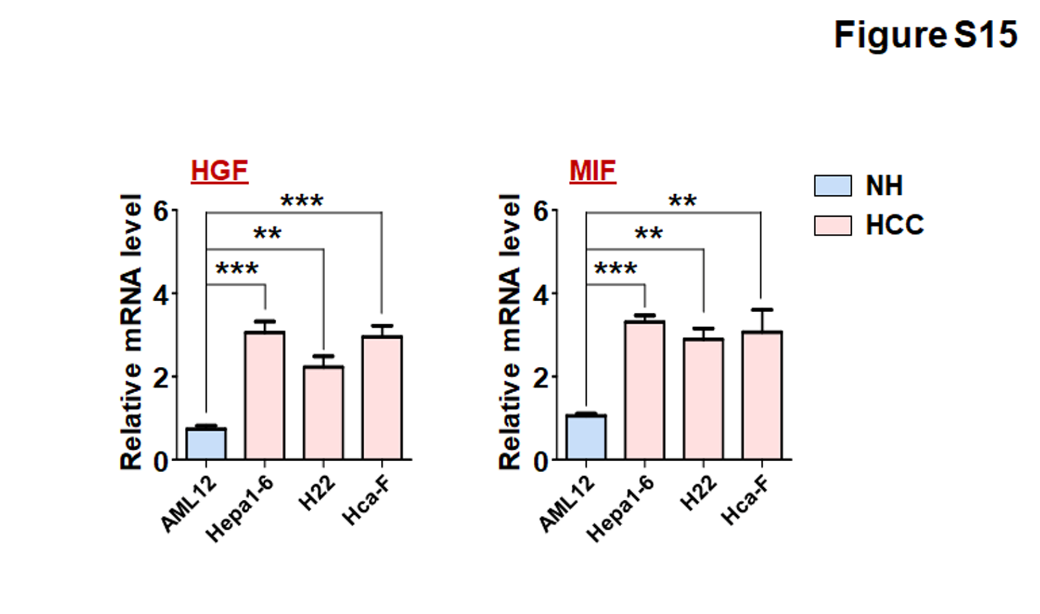


**Figure S15. qRT-PCR assay for the expression of HGF and MIF in normal hepatic (NH) and HCC cell lines (n = 6).**


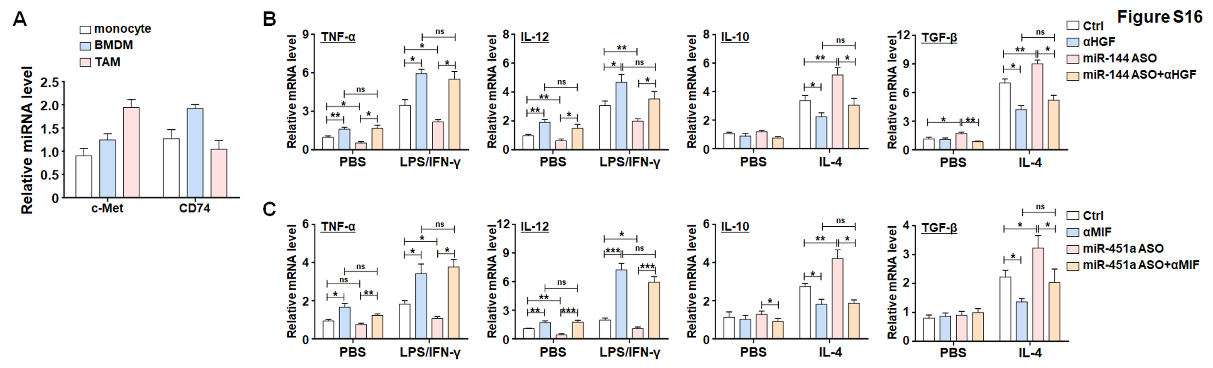


**Figure S16. miR-144/451a in HCC cells regulate macrophage polarization through paracrine HGF and MIF.** (A) Monocytes and TAMs were sorted from bone marrow (BM) and tumor tissue, respectively. BMDMs were stimulated from BM cells with M-CSF (25 ng/ml) for 7 days. The expression of HGF receptor (c-Met) and MIF receptor (CD74) was determined in these cells. (B) BMDMs were cultured from bone marrow cells via M-CSF stimulation. Hepa1-6 cells were transfected with miR-144 ASO or/and incubated with HGF antibody (αHGF), and the supernatant was used for culture of stimulated BMDMs. The expression of indicated genes in macrophages was determined by qRT-PCR (n = 5). (C) Hepa1-6 cells were transfected with miR-451a ASO or/and incubated with MIF antibody (αMIF), and the supernatant was used for culture of stimulated BMDMs. The expression of indicated genes in macrophages was determined by qRT-PCR (n = 5). Bars, means ± SEMs; *, P < 0.05; **, P < 0.01; ***, P < 0.001; ns, not significant.


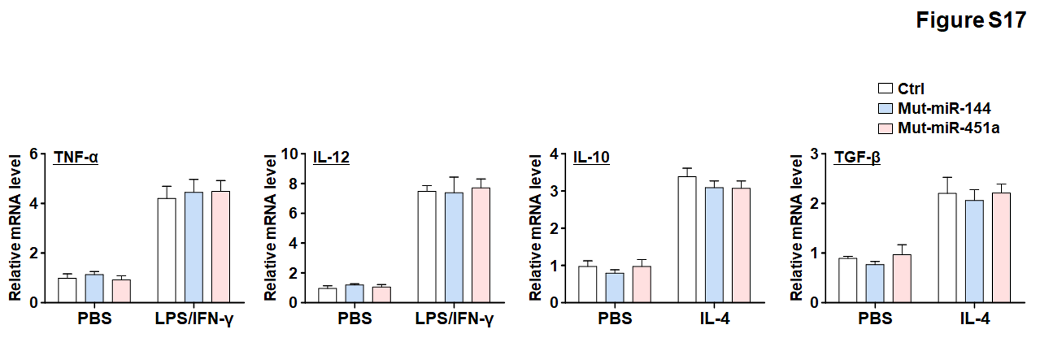


**Figure S17. Mutant miR-144/miR-451a expression in HCC cells fails to affect the polarization of cocultured BMDMs.** Hepa1-6 cells were modified to overexpress seed sequence-mutated miR-144 (Mut-miR-144) or mutated miR-451a (Mut-miR-451a), and the supernatant was prepared to culture BMDMs with the indicated stimuli. The function of macrophages was determined by qRT-PCR (n = 5). Bars, means ± SEMs.


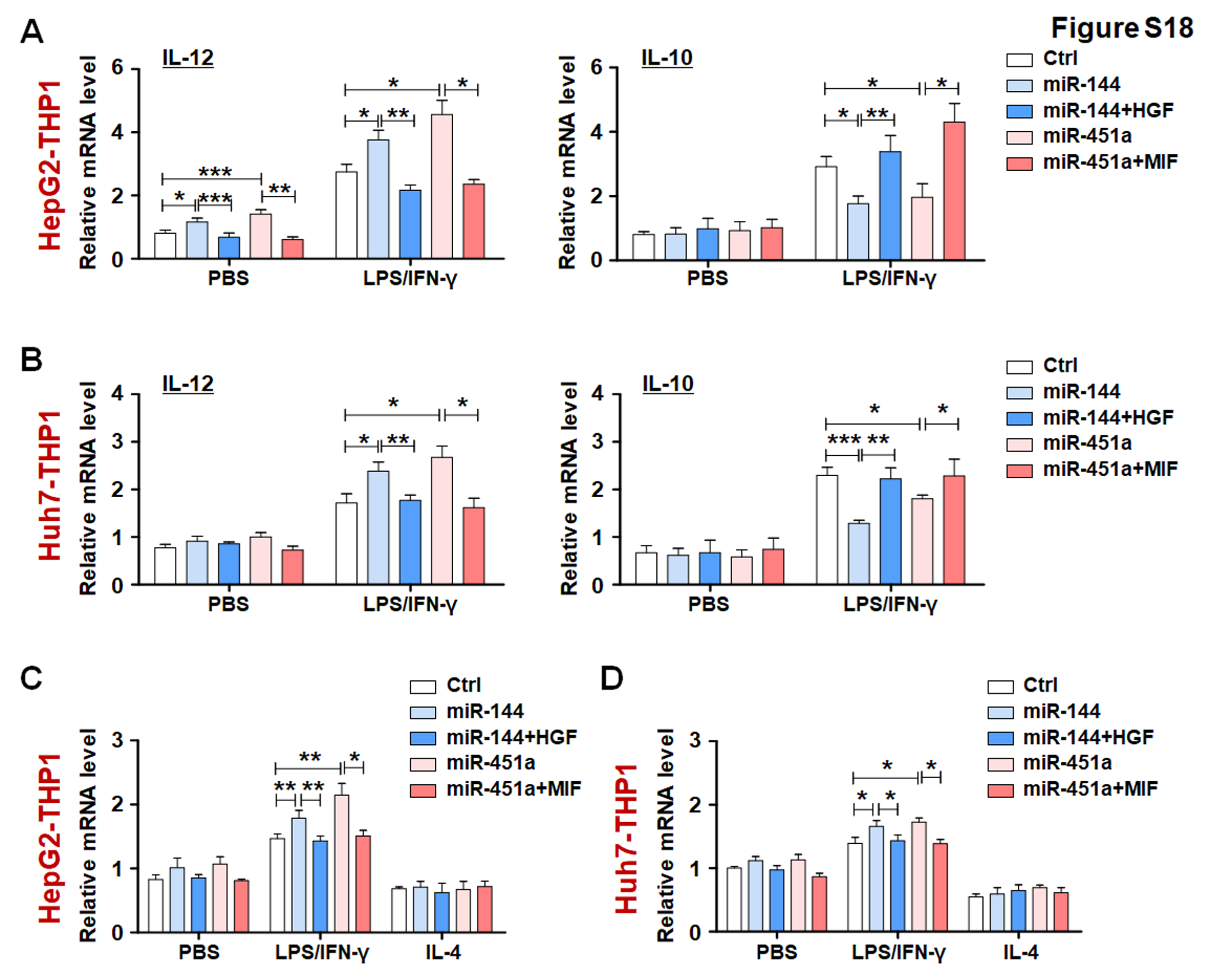


**Figure S18. miR-144/miR-451a in human HCC cells regulate the function of cocultured human macrophages by targeting HGF and MIF.** (A, B) HepG2 (A) and Huh7 (B) cells were modified to overexpress miR-144 or miR-451a, and human recombinant HGF (20ng/ml) or MIF (20ng/ml) was added as indicated. The supernatant was harvested and used for culture of stimulated human macrophage cell line, THP1 (n = 5). (C, D) THP1 cells were cultured as described in (A, B), and the NO generation was measured (n = 5). Bars, means ± SEMs; *, P < 0.05; **, P < 0.01; ***, P < 0.001.


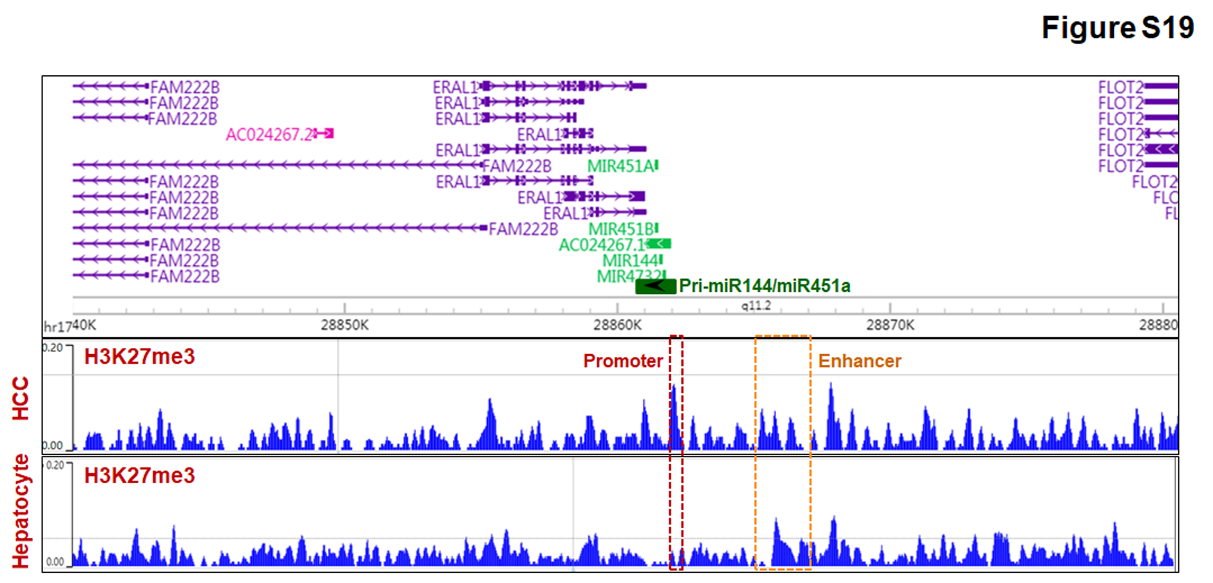


**Figure S19. The potential H3K27me3 modification in the promoter and enhancer regions of pri-miR-144/451a were analyzed using ChIP-seq data of hepatocytes or HCC cells from the Cistrome Project.**


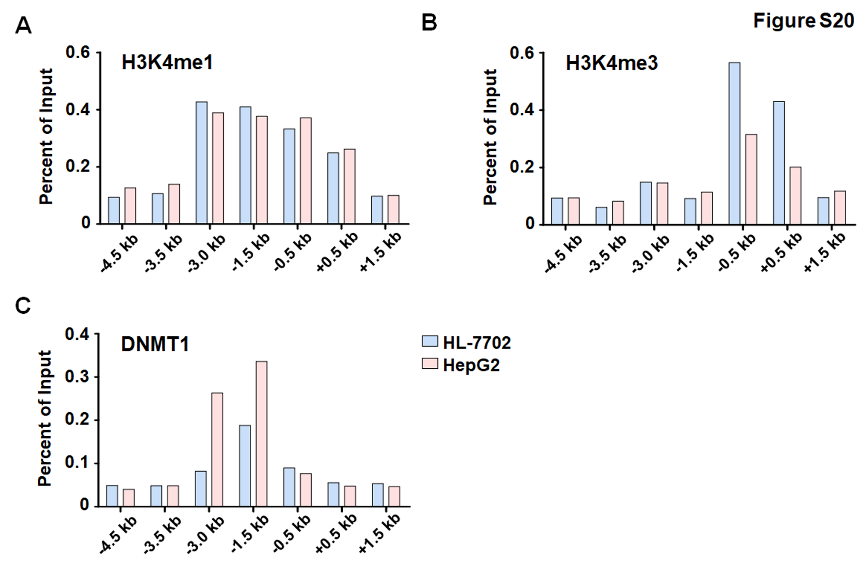


**Figure S20. Enhanced DNMT1 enrichment and regressed promoter activity on the pri-miR-144/451a locus in HCC cell lines.** (A-C) ChIP assays were performed using lysates of HepG2 cells with antibodies of H3K4me1, H3K4me3 and DNMT1. The different fragments of the Pri-miR-144/miR-451a promoter were amplified and compared between the two cell lines.


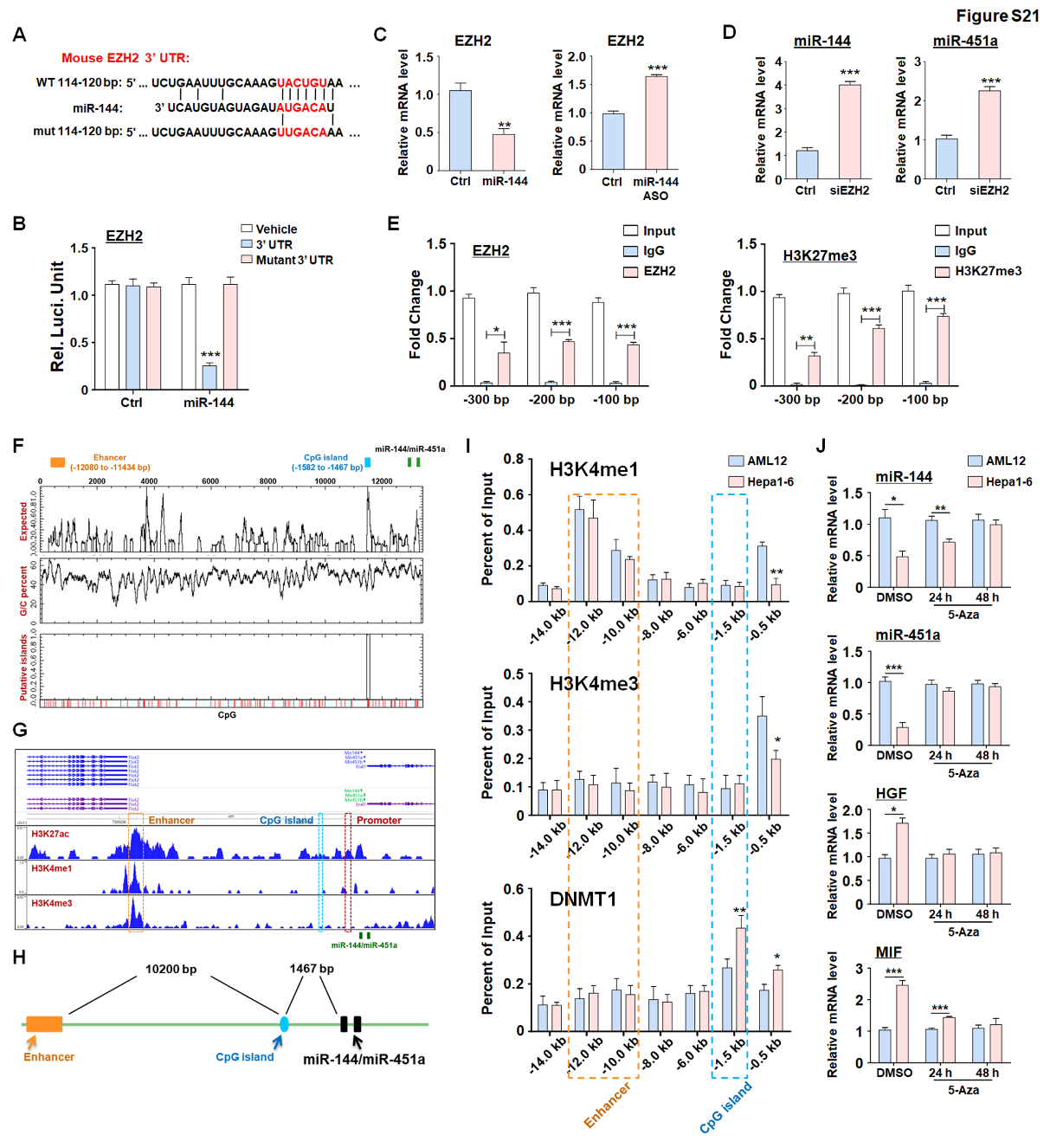


**Figure S21.** **DNA methylation-dependent chromatin remodeling regulates miR-144/451a cluster in mouse HCC cells.** (A, B) Mouse EZH2 was also identified as a target of mouse miR-144, which was validated by an assay showing that miR-144 inhibited luciferase expression from a construct harboring a 3’UTR of mouse *EZH2* (n = 6). (C) The mRNA level of EZH2 was detected in miR-144-overexpressing or miR-144-inhibited Hepa1-6 cells (n = 6). (D) Hepa1-6 cells were subjected to EZH2 knockdown, followed by qRT-PCR assay for the expression of miR-144 and miR-451a (n = 6). (E) ChIP assays were performed using antibodies of EZH2 or H3K27me3 in Hepa1-6 cell lysates to further determine the miR-144/451a promoter sites involved in EZH2 binding and H3K27me3 modification (n = 4). (F) The sequence of mouse chromatin region covering the miR-144/451a locus and the 5’-flanking sequences was analyzed, and the CpG islands were predicted. (G) ChIP-seq data from the Cistrome Project were used to analyze sites potentially bound by H3K27ac, H3K4me1 and H3K4me3 on the miR-144/451a locus. (H) A diagram showing the relative position of mouse miR-144/451a, the predicted CpG island and the enhancer region. (I) ChIP assays were performed using antibodies of H3K4me1, H3K4me3 and DNMT1 in lysates of AML12 and Hepa1-6 cells. Different 5’ flanking regions of the mouse miR-144/miR-451a promoter were amplified and compared (n = 4). (J) AML12 and Hepa1-6 cells were treated with a DNMT1 inhibitor (5-Aza) or DMSO. The expression of the indicated miRNA transcripts and targets was determined via qRT-PCR (n = 5). Bars, means ± SEMs; *, P < 0.05; **, P < 0.01; ***, P < 0.001.


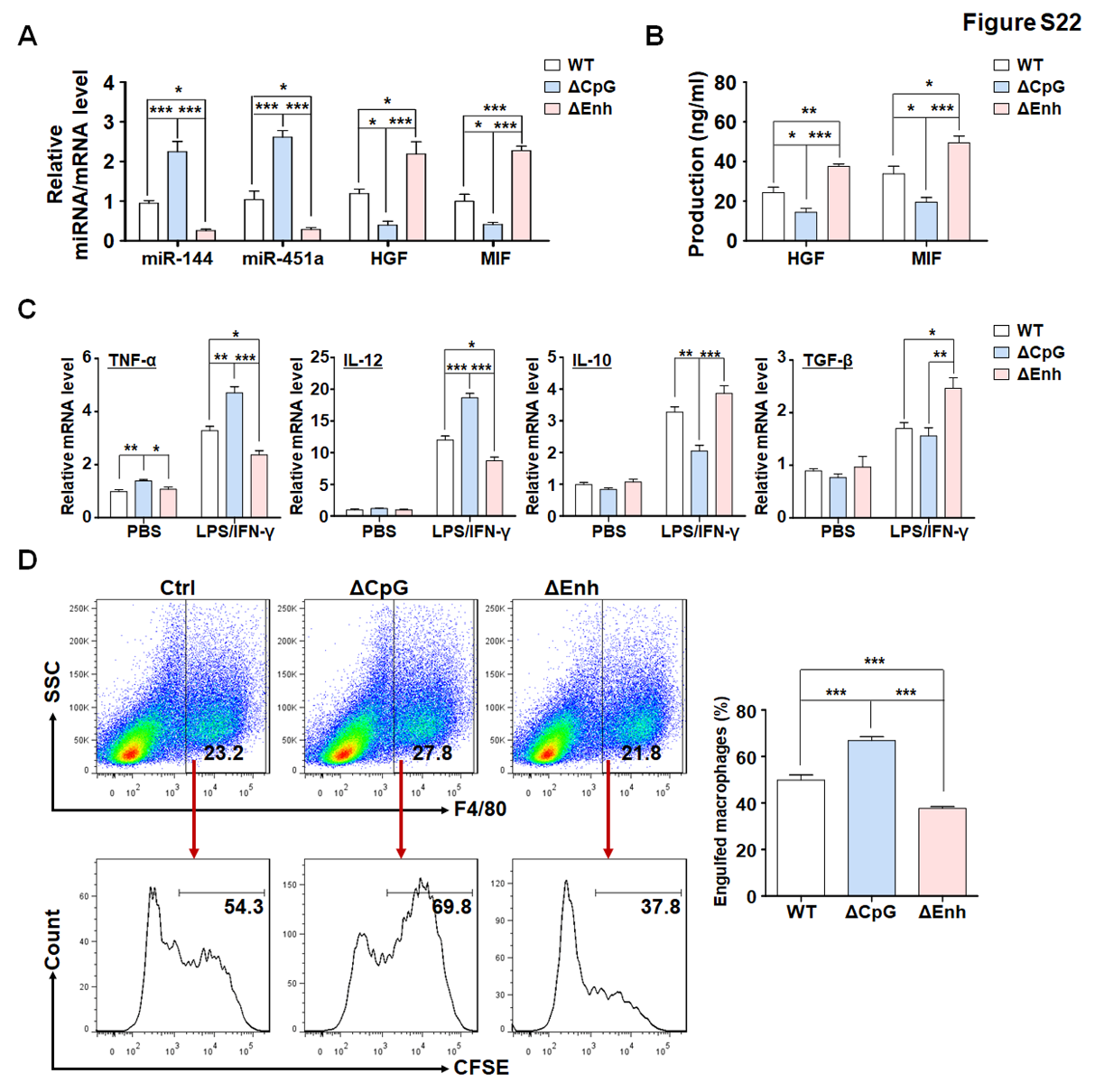


**Figure S22. miR-144/451a upstream CpG island or enhancer deletion in HCC cells modulates macrophage function via paracrine factors.** (A) The CpG island or the enhancer region upstream of the miR-144/451a locus was depleted in HCC cells and the expression of miR-144/miR-451a and their targets were determined via qRT-PCR (n = 5). (B) The supernatant of CpG island or enhancer knockout cells was harvested to measure the production of HGF and MIF via ELISA (n = 5). (C) The supernatant of CpG island or enhancer knockout cells was harvested and used for culture of macrophages, and the levels of polarization markers were assayed by qRT-PCR (n = 5). (D) The stimulated macrophages in (C) were cocultured with CFSE-stained Hepa1-6 cells. Tumor cell engulfment by macrophages was detected via FACS (n= 6). Bars, means ± SEMs; *, P < 0.05; **, P < 0.01; ***, P < 0.001.


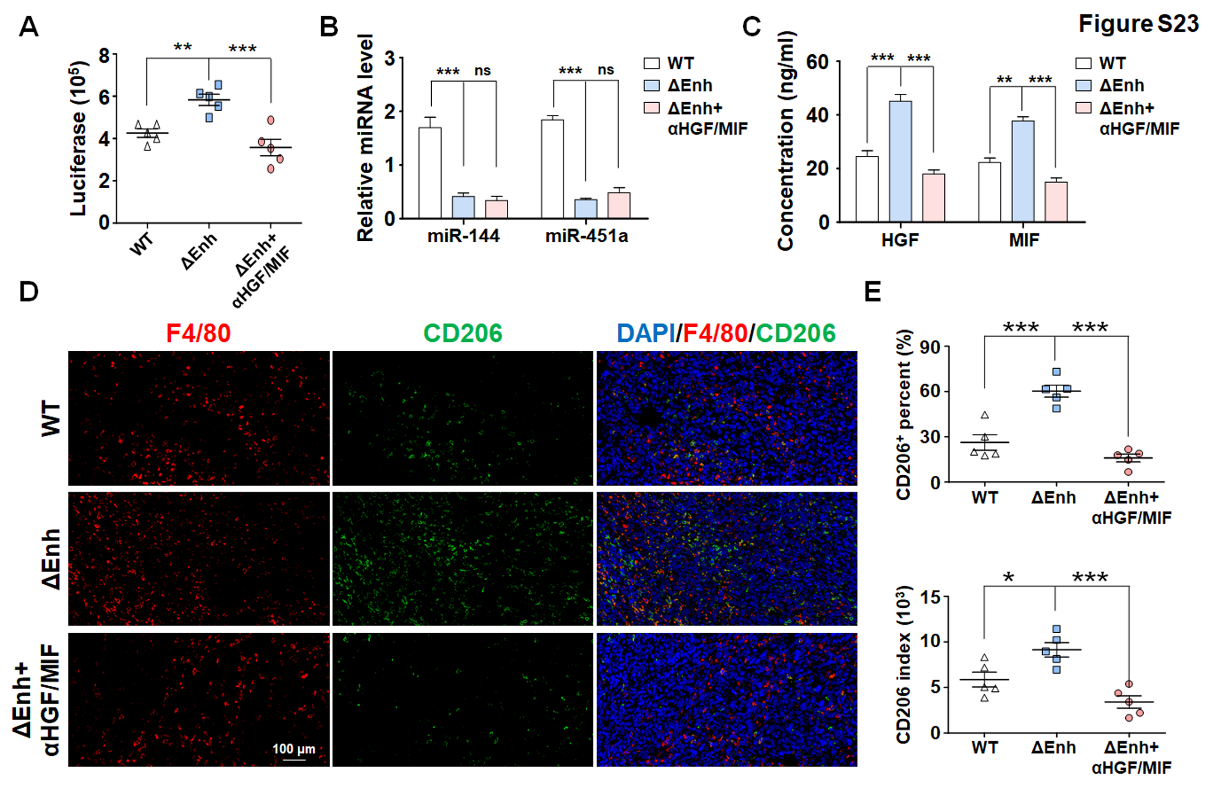


**Figure S23. Blocking HGF and MIF using neutralizing antibodies relieved the effect of pri-miR-144/451a enhancer deletion in vivo.** (A) The enhancer region upstream to miR-144/451a was deleted via CRISPR/cas9 system in luciferase-expressing Hepa1-6 cells, and cells were inoculated intrahepatically into C57BL/6 mice with or without combination antibodies of HGF and MIF. Tumor development was evaluated by bioluminescence imaging three weeks after the injection (n = 5). (B) The expression of miR-144/miR-451a in tumor tissues was measured by qRT-PCR (n = 5). (B) The secretion of HGF and MIF in the serum was measured via ELISA (n = 5). (D, E) Tumor tissues were co-stained with F4/80 and CD206 and analyzed for expression of the M2-like macrophage marker, CD206 (n = 5). Bars, means ± SEMs; *, P < 0.05; **, P < 0.01; ***, P < 0.001.
